# Supplementary material for: Upcycling of atmospheric CO2 to self-healing recyclable polymers under ambient conditions
Source: Nat Commun. 2026 Mar 2;17:3349. doi: 10.1038/s41467-026-70046-6 (PMC13066383; doi:10.1038/s41467-026-70046-6)
Supplement: Supplementary file 1 — Supplementary Information [file 41467_2026_70046_MOESM1_ESM.pdf]

Supplementary Information for

Upcycling of Atmospheric CO_2 to Self-Healing Recyclable Polymers under Ambient Conditions

Xiaoyue Zeng[1§], Shiguang Zhang[1§], Huiya Li[2§], Chun Liu[1] & Liang Chen[1*]

[1]Chongqing Key Laboratory of Soft-Matter Material Manufacturing, School of Chemistry and Chemical Engineering, Southwest University, Chongqing, China.

[2]Analytical & Testing Center, Southwest University, Chongqing, China.

[§]These authors contributed equally: Xiaoyue Zeng, Shiguang Zhang, Huiya Li.

*Corresponding author. e-mail: liangchen16@swu.edu.cn

This file includes:

Supplementary Figs. 1-81

Supplementary Tables 1-2

Supplementary References

Other Supplementary Materials for this manuscript include the following:

Supplementary Movies 1-3

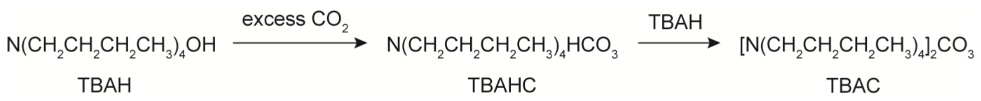

Supplementary Fig. 1 | Synthetic route for TBAC.

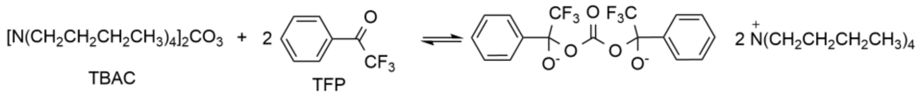

Supplementary Fig. 2 | TFP-CO_3^{2-} reaction.

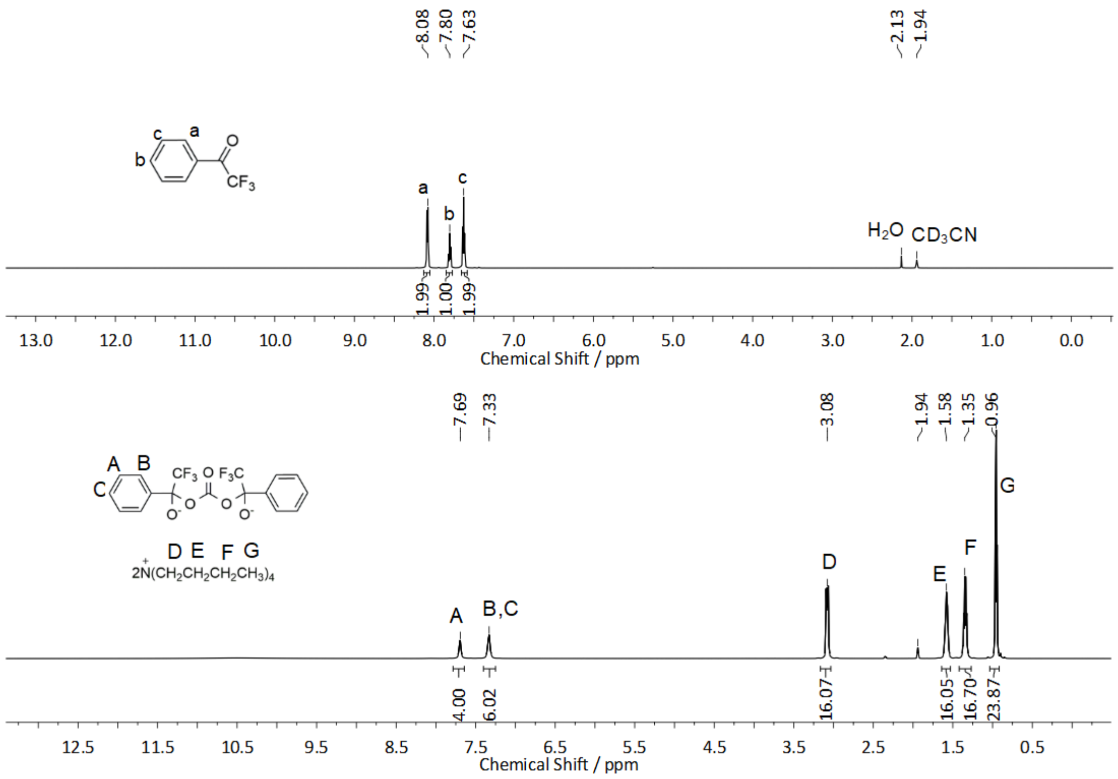

Supplementary Fig. 3 | ^{1}H NMR spectrum (in CD_3CN) of TFP (top) and the CO_3^{2-}-bridged product TFPCTFP (bottom). The reaction was nearly quantitatively complete.

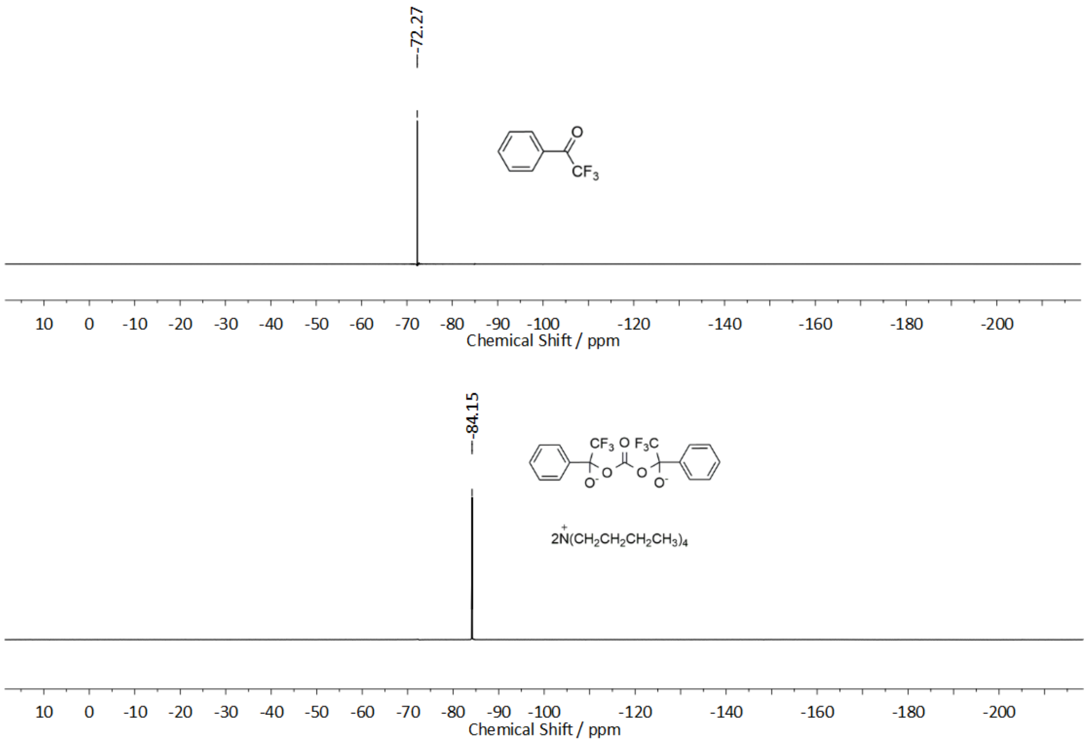

Supplementary Fig. 4 | ^{19}F NMR spectrum (in CD3CN) of TFP (top) and the CO_3^{2-}-bridged product TFPCTFP (bottom).

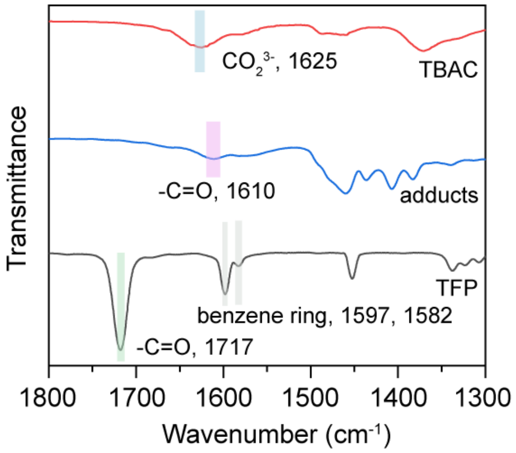

Supplementary Fig. 5 | FTIR spectroscopy of TFP, TBAC and CO_3^{2-}-bridged adducts. As the reaction progresses, the carbonyl peak of TFP at 1717 cm^{-1} and the carbonate peak of TBAC at 1625 cm^{-1} disappear completely, concomitantly with the appearance of a weak new peak at 1610 cm^{-1}, which is assigned to the carbonyl stretch of the CO_3^{2-}-bridged adduct. Additionally, the intensity of the benzene ring absorption decreases significantly, likely because the adduct's electrostatic field reduces the vibrational dipole moment.

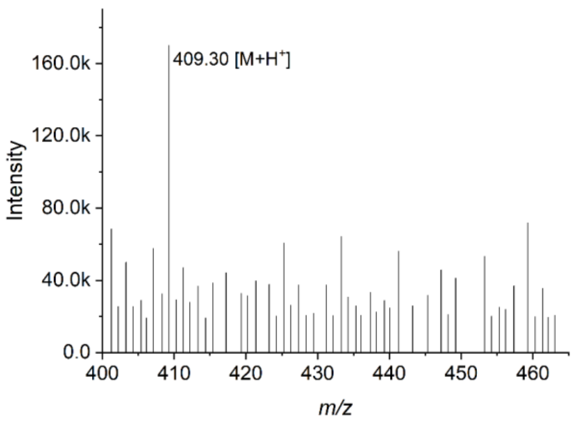

Supplementary Fig. 6 | Mass spectra of TFPCTFP.

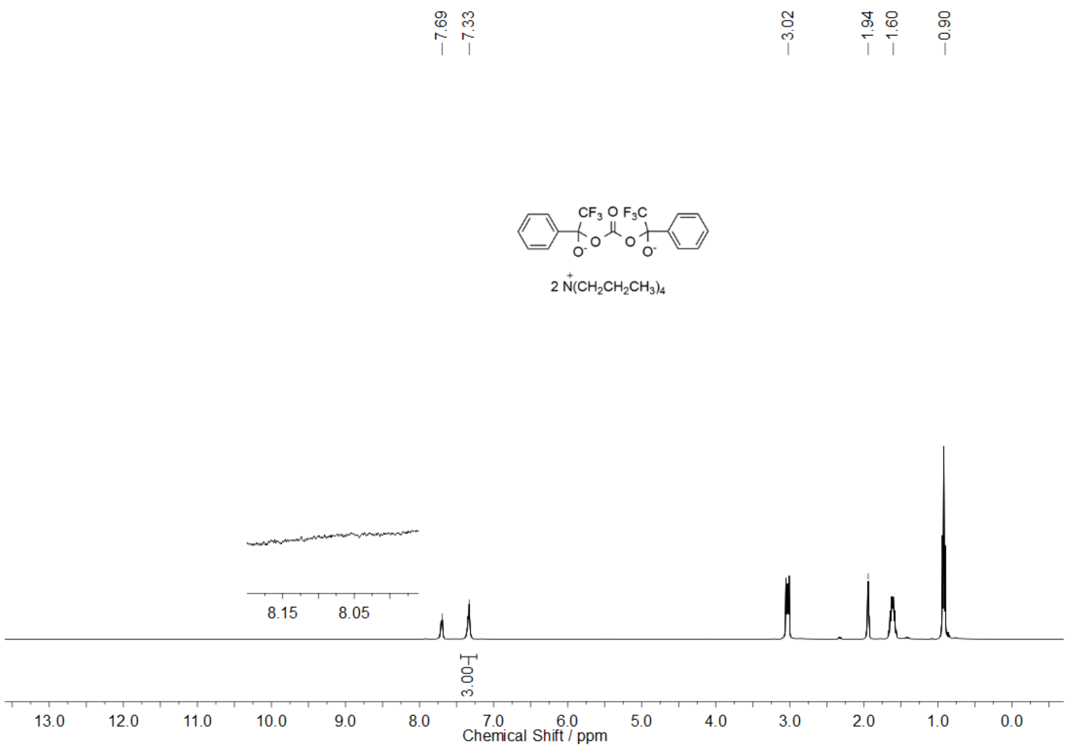

Supplementary Fig. 7 | ^{1}H NMR spectrum (in CD_3CN) of TPAC-TFP reaction. The reaction was nearly quantitatively complete.

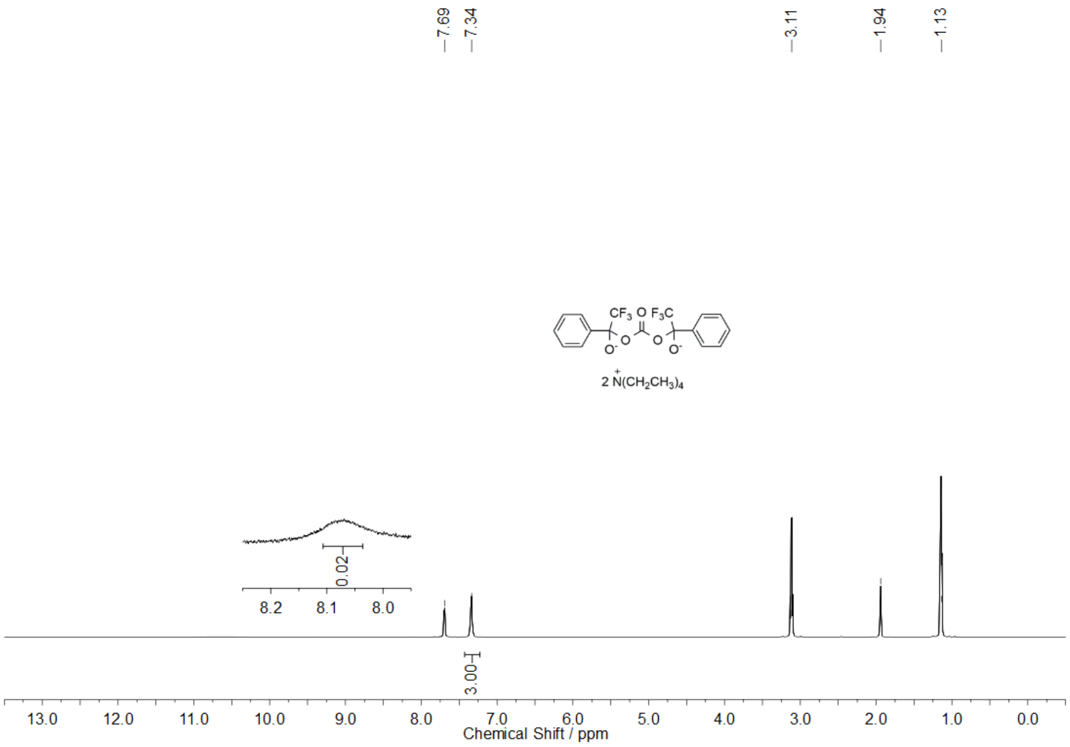

Supplementary Fig. 8 | ^{1}H NMR spectrum (in CD_3CN) of TEAC-TFP reaction. The yield was calculated to be 99.0%.

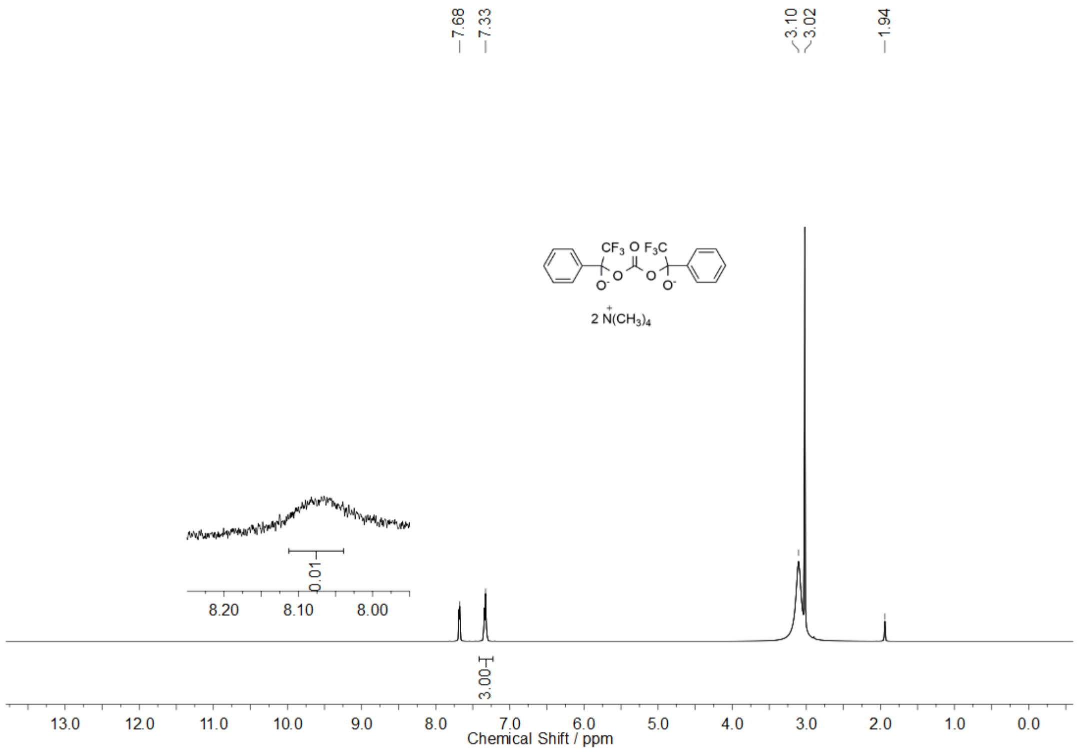

Supplementary Fig. 9 | ^{1}H NMR spectrum (in CD_3CN) of TMAC-TFP reaction.

The yield was calculated to be 99.5%.

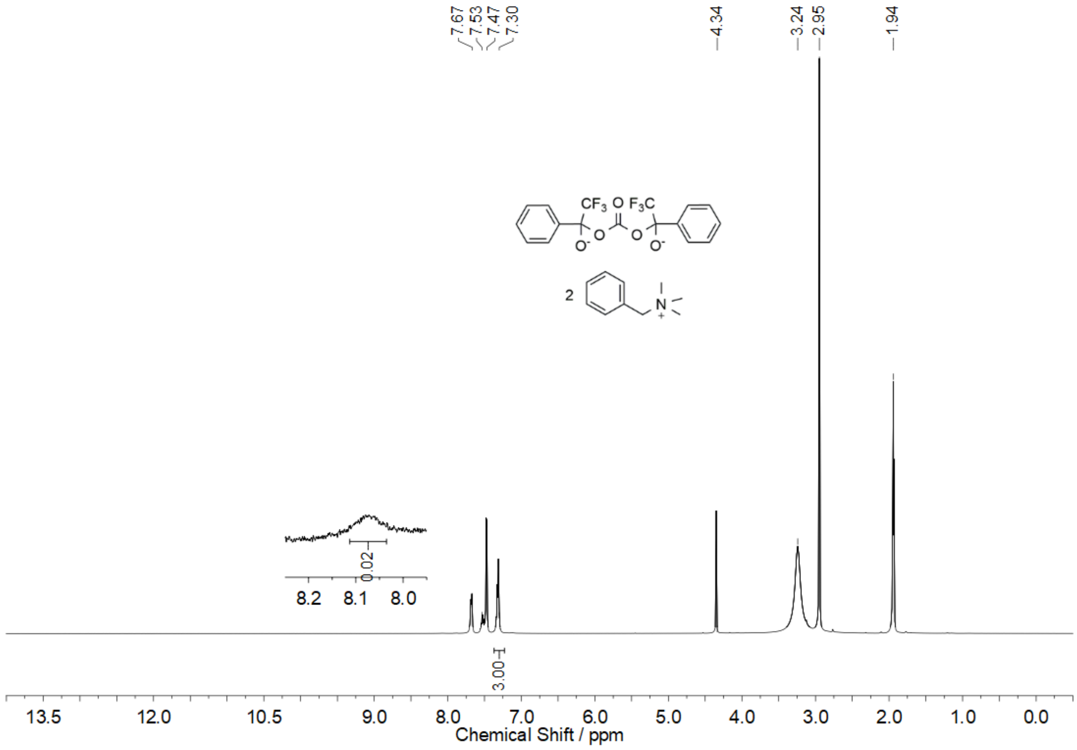

Supplementary Fig. 10 | ^{1}H NMR spectrum of BnMAC-TFP reaction (in CD_3CN).

The yield was calculated to be 99.0%.

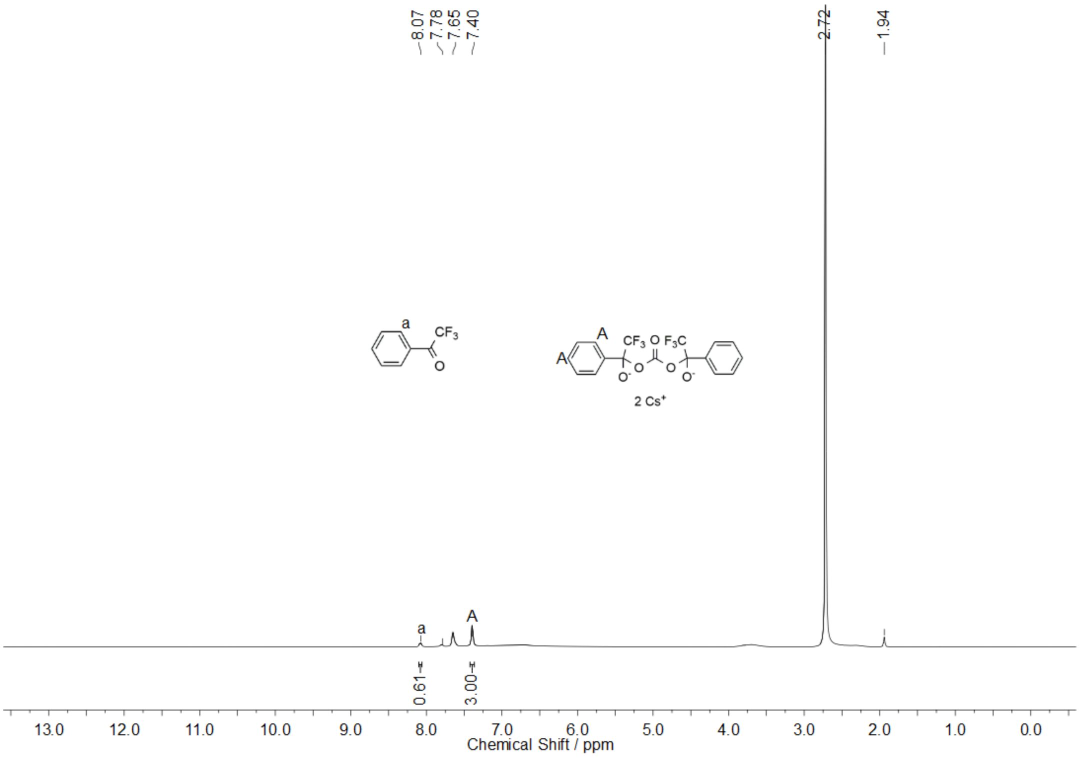

Supplementary Fig. 11 | ^{1}H NMR spectrum (in CD_3CN) of Cs_2CO_3-TFP reaction.

The yield was calculated to be 76.6%.

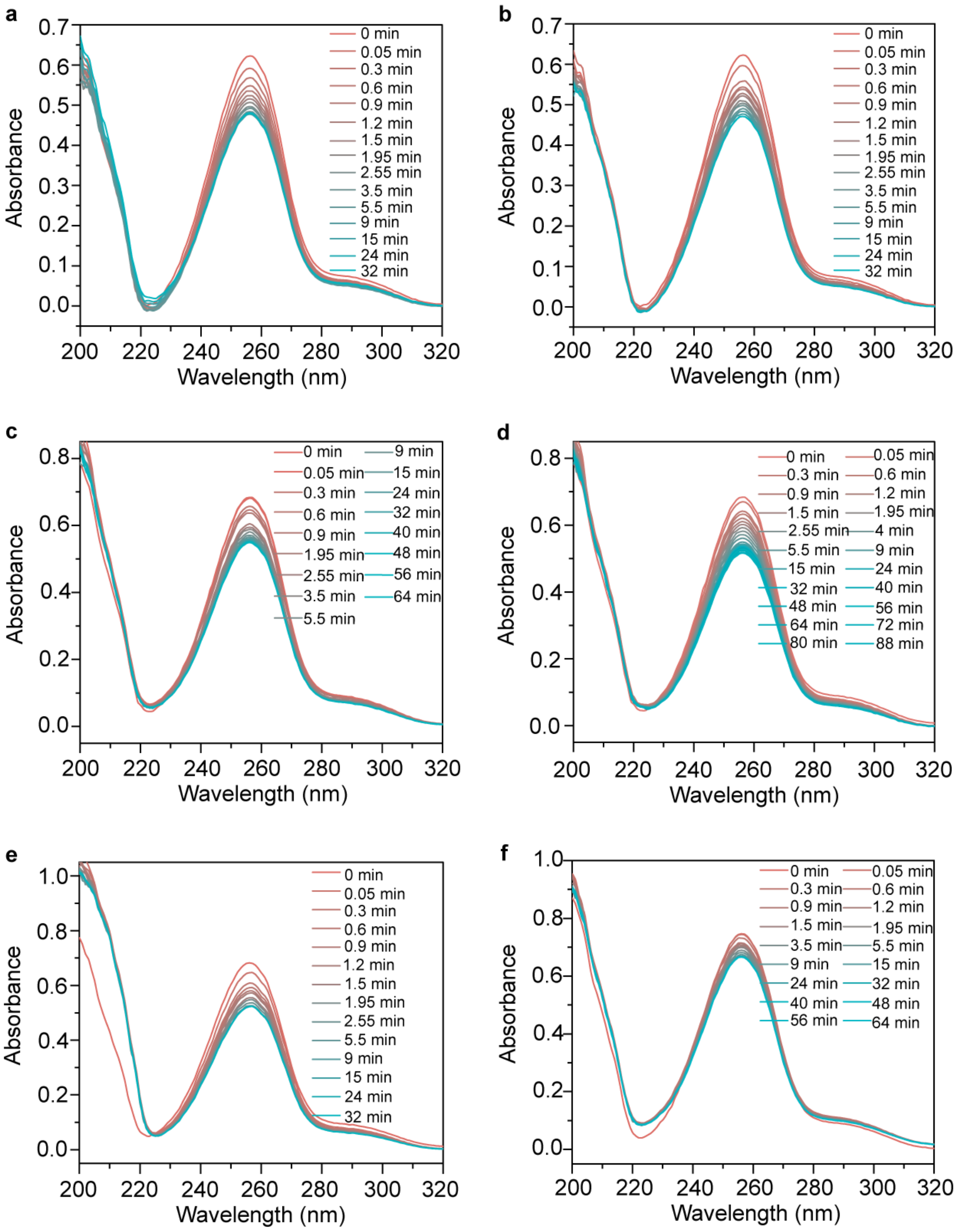

Supplementary Fig. 12 | UV-Vis spectroscopy for monitoring the TFP-CO_3^{2-} reaction with different carbonate salts. a, TBAC; **b**, TPAC; **c**, TEAC; **d**, TMAC; **e**, BnMAC; **f**, Cs_2CO_3.

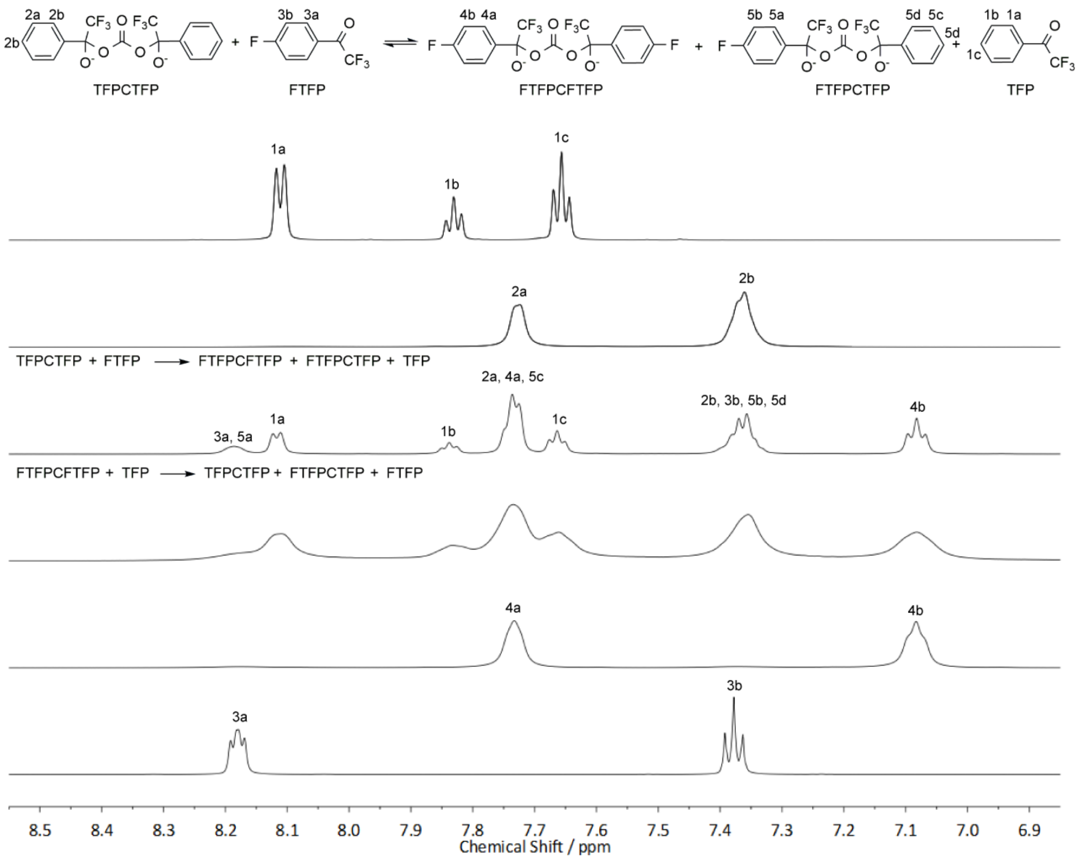

Supplementary Fig. 13 | ^{1}H NMR spectra (in CD_3CN) (from top to bottom) of TFP, TFPCTFP, equilibrium state of exchange reaction started from TFPCTFP and FTFP, equilibrium state of exchange reaction started from FTFPCFTFP and TFP, FTFPCFTFP, FTFP.

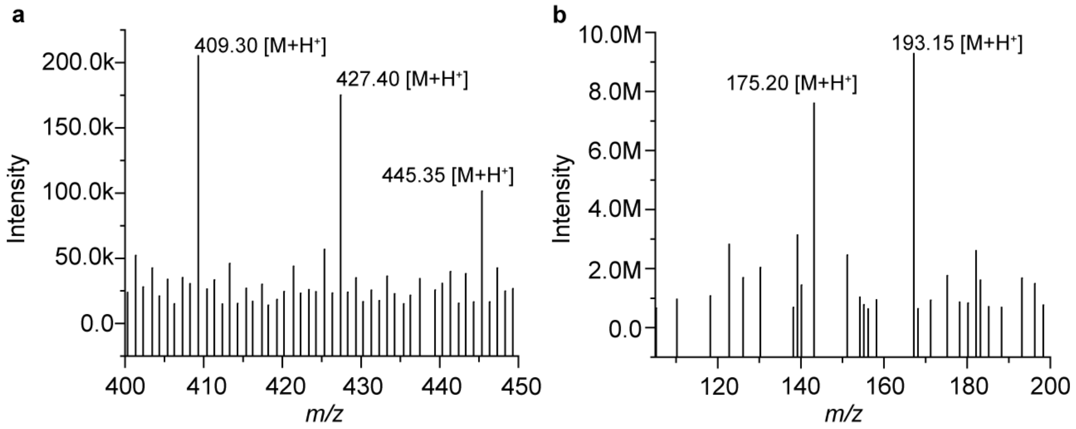

Supplementary Fig. 14 | Representative mass spectra of exchange reaction products. Two independent, representative measurements are shown, which together confirm the generation of all five expected products. **a**, Mass spectrum from one trial, in which signals corresponding to TFPCTFP, TFPCFTFP and FTFPCFTFP. b, Mass spectrum from a separate trial, in which signals for TFP and FTFP. Detected *m/z* values (from left to right): TFPCTFP ($C_{17}H_{11}F_6O_5$ [M+H$^+$], calcd 409.26, found 409.30), TFPCFTFP ($C_{17}H_{10}F_7O_5$ [M+H$^+$], calcd 427.25, found 427.40), FTFPCFTFP ($C_{17}H_9F_8O_5$ [M+H$^+$], calcd 445.24, found 445.35), TFP ($C_8H_6F_3O$ [M+H$^+$], calcd 175.13, found 175.20), FTFP ($C_8H_5F_4O$, calcd 193.12, found 193.15).

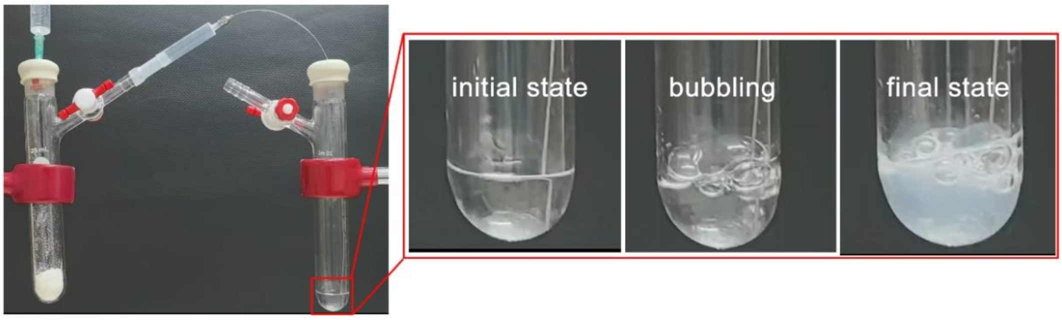

Supplementary Fig. 15 | Evidence of CO_2 production during acid-induced dissociation. As the reaction progressed, vigorous gas evolution was observed, accompanied by the gradual opacification of the initially transparent $Ca(OH)_2$ aqueous solution.

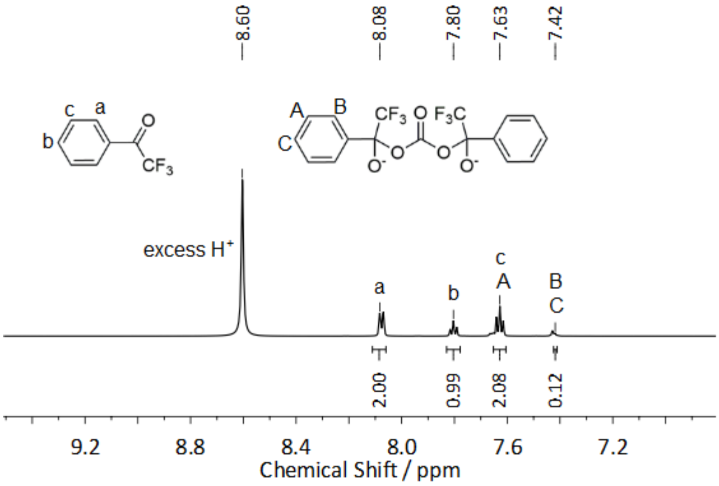

Supplementary Fig. 16 | ^1H NMR spectra (in CD_3CN) of the acid-induced dissociation reaction. The yield was calculated to be 96.2%.

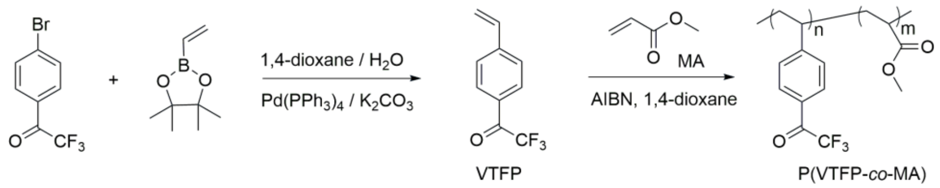

Supplementary Fig. 17 | Synthetic route for P(VTFP-MA)-20.

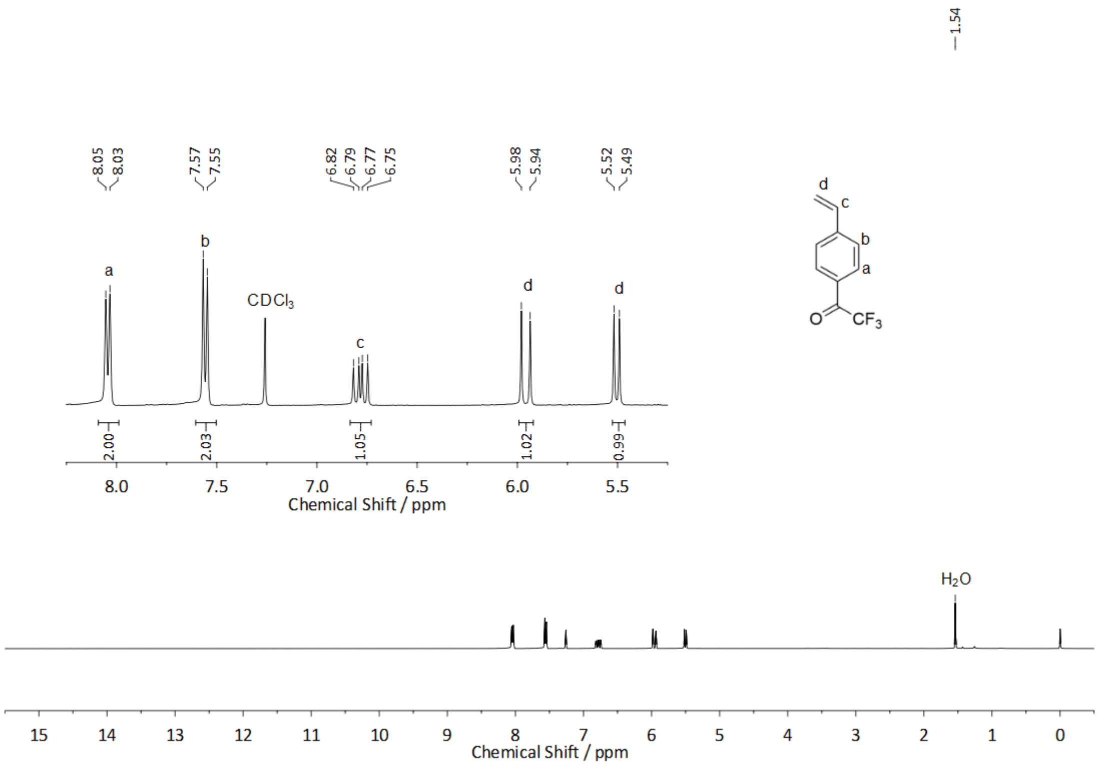

Supplementary Fig. 18 | ^{1}H NMR spectrum (in $CDCl_3$) of VTFP.

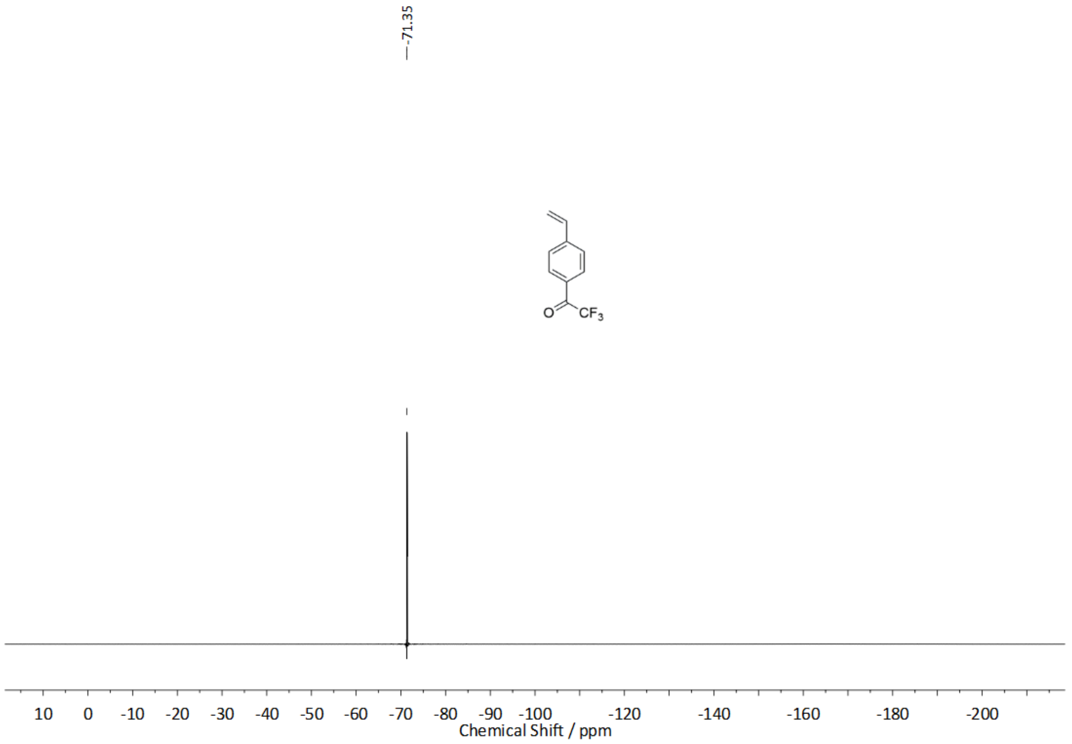

Supplementary Fig. 19 | ^{19}F NMR spectrum (in $CDCl_3$) of VTFP.

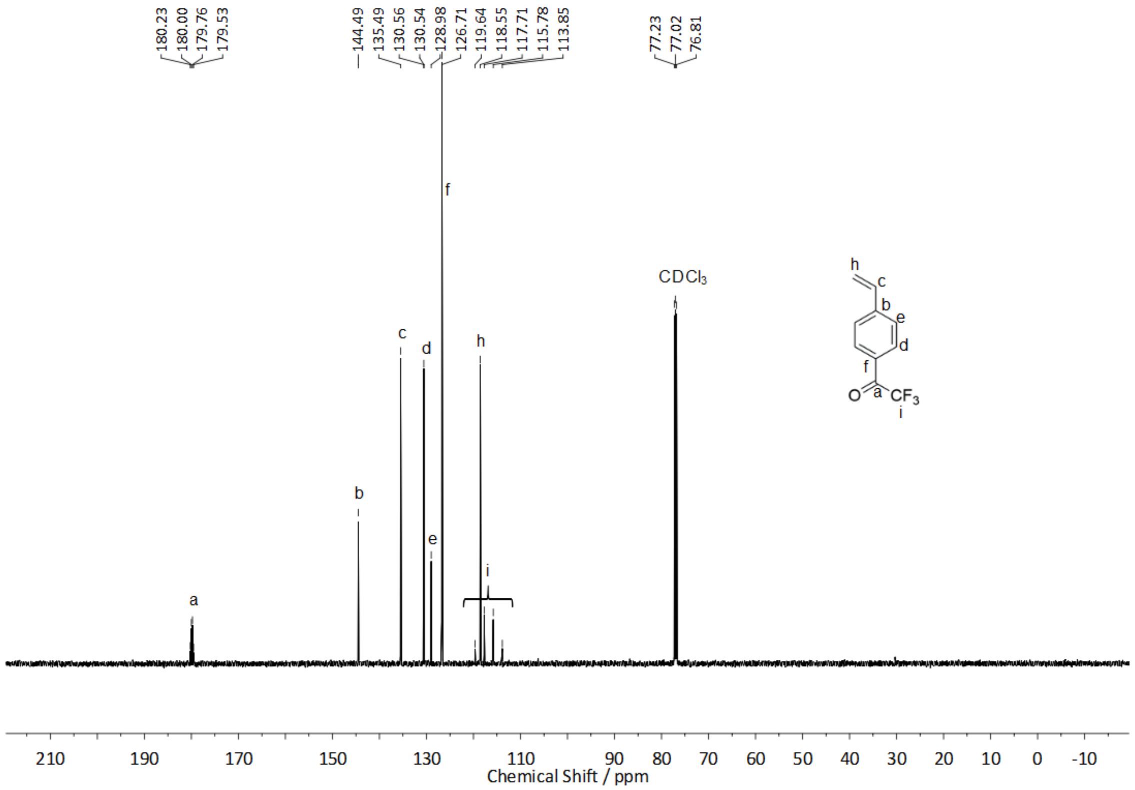

Supplementary Fig. 20 | ^{13}C NMR spectrum (in $CDCl_3$) of VTFP.

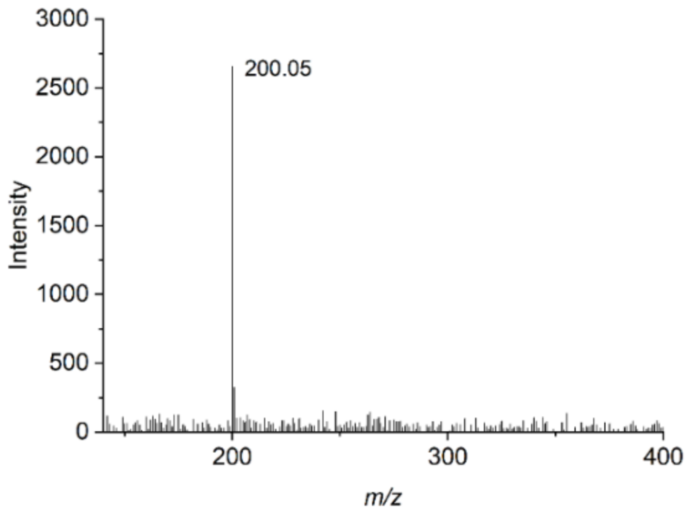

Supplementary Fig. 21 | Mass spectrum of VTFP.

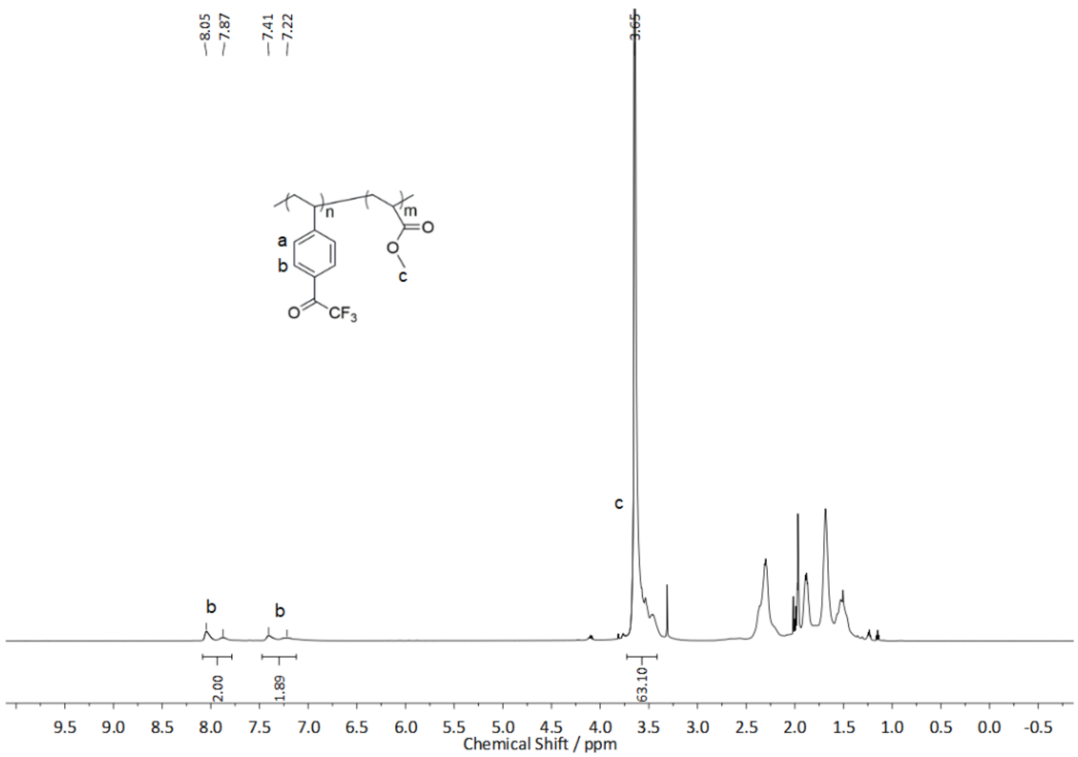

Supplementary Fig. 22 | ^{1}H NMR spectrum (in CD_3CN) of liner copolymer P(VTFP-MA)-20.

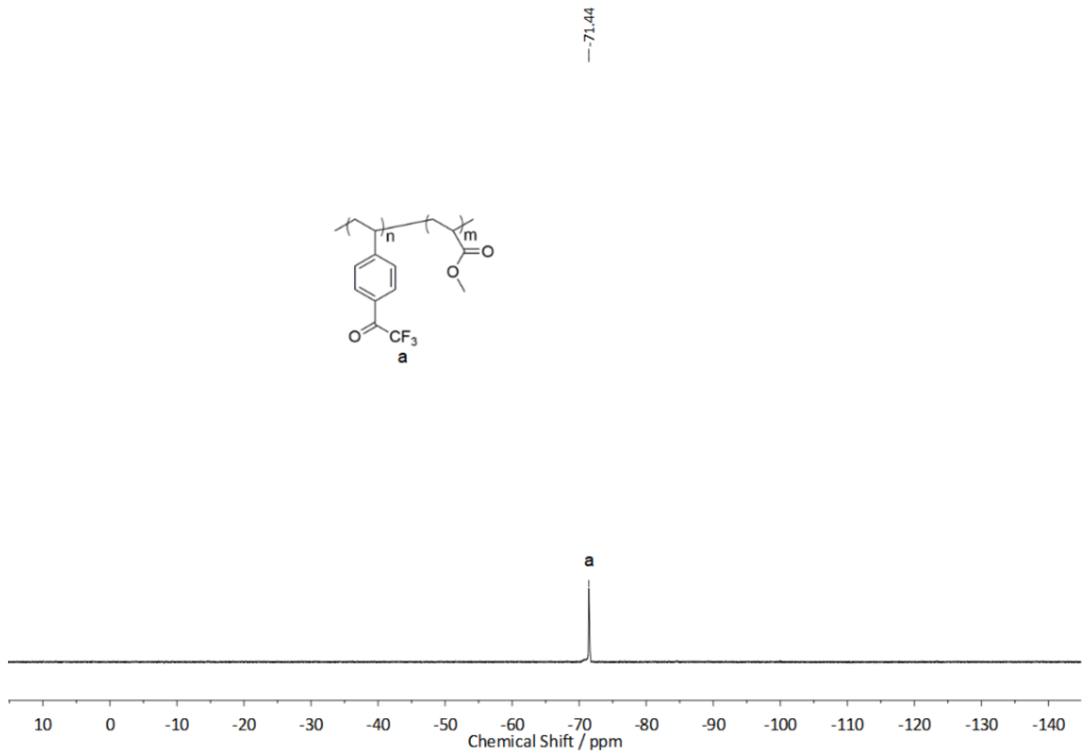

Supplementary Fig. 23 | ^{19}F NMR spectrum (in CD_3CN) of liner copolymer P(VTFP-MA)-20.

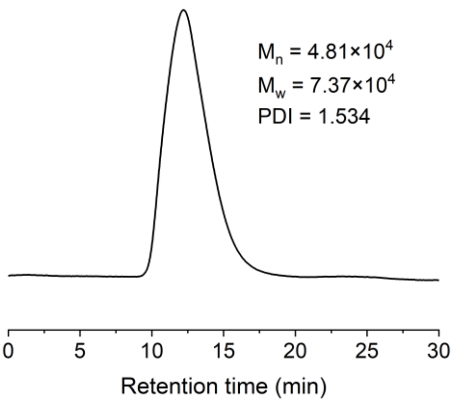

Supplementary Fig. 24 | GPC spectrum of the liner copolymer P(VTFP-MA)-20 using THF as the eluent.

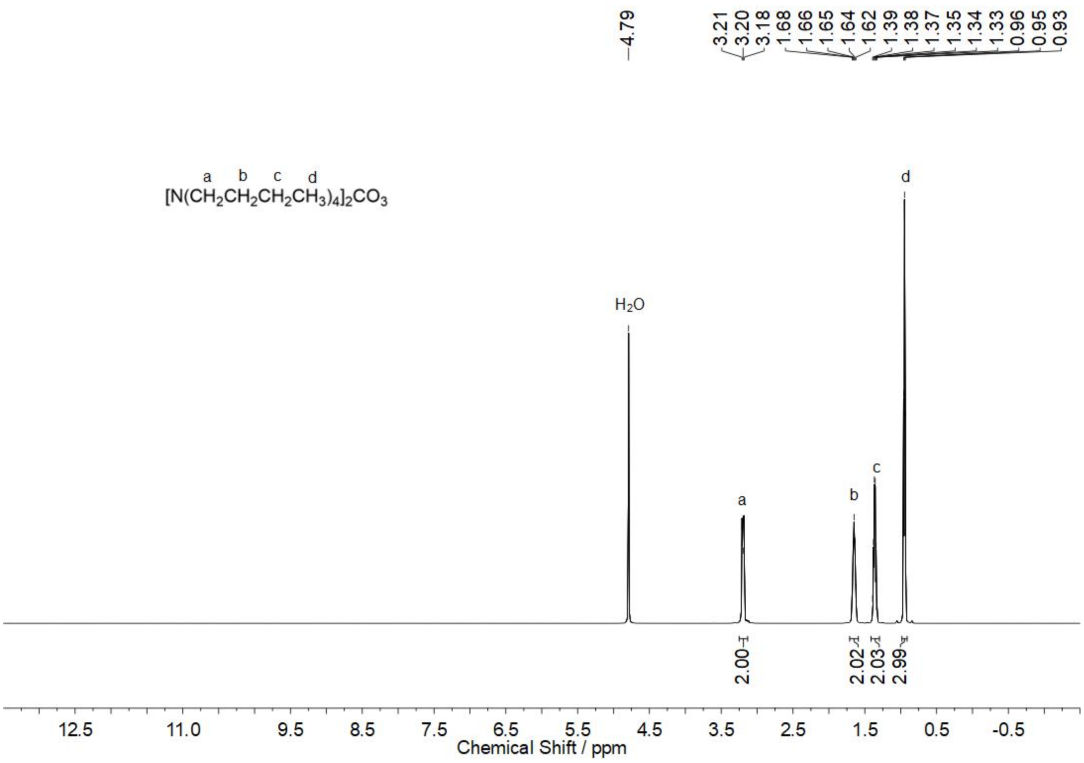

Supplementary Fig. 25 | ^{1}H NMR spectrum (in D_2O) of TBAC derived from atmospheric CO_2.

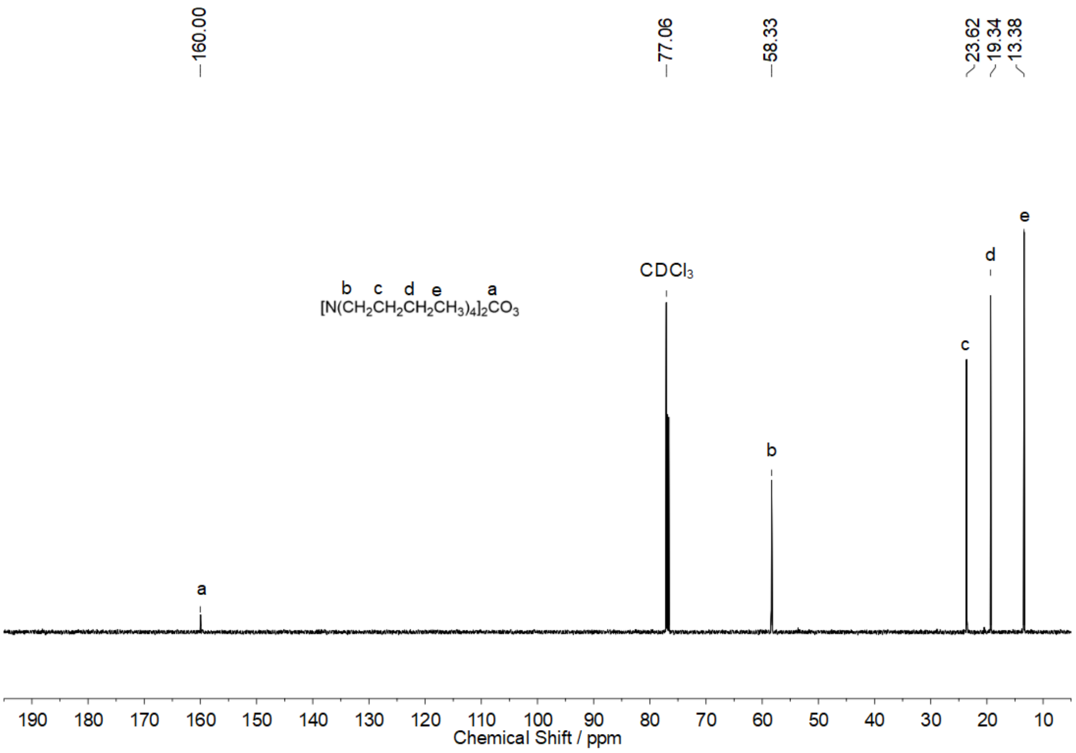

Supplementary Fig. 26 | ^{13}C NMR spectrum (in $CDCl_3$) of TBAC derived from atmospheric CO_2.

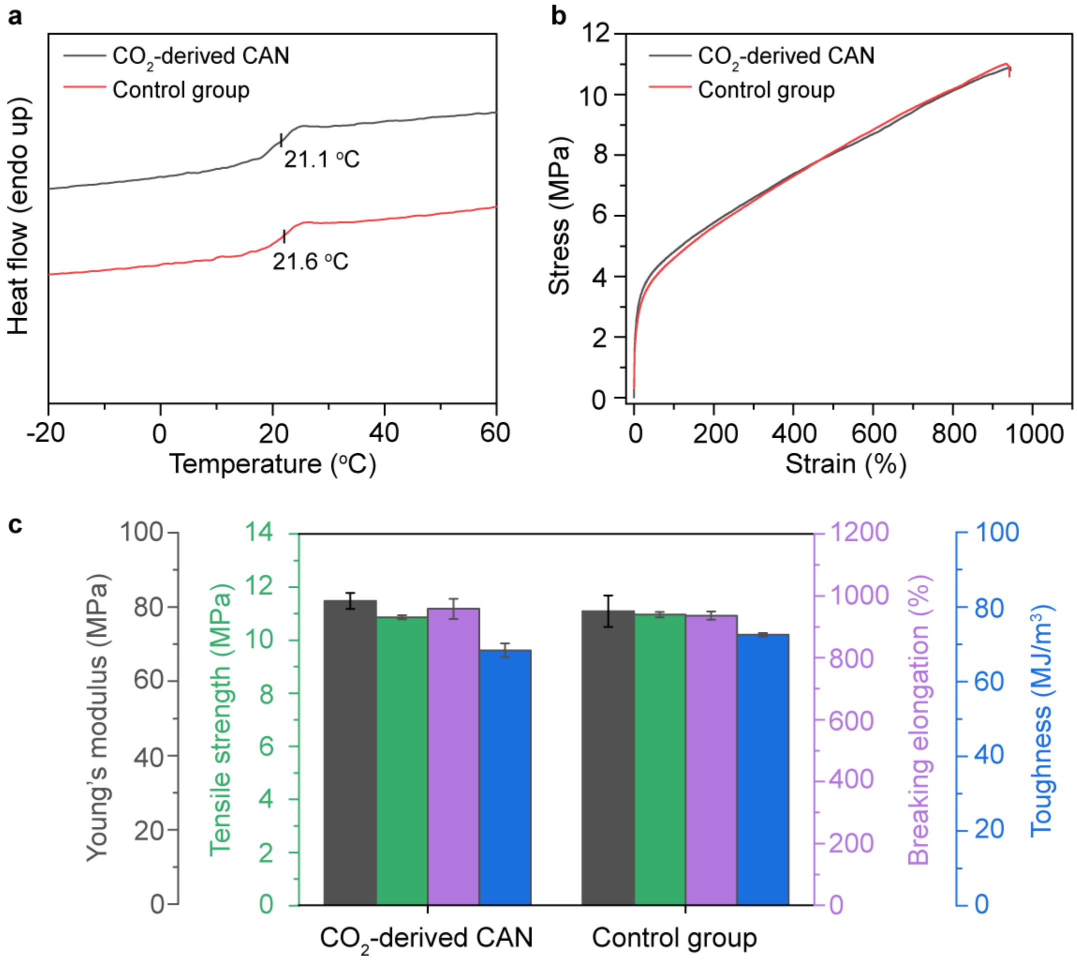

Supplementary Fig. 27 | Comparison of thermal and mechanical properties between the CO_2-derived CAN and the network cross-linked with pure TBAC. **a**, DSC curves. **b**, Representative tensile stress-strain curves. **c**, Comparison of Young's modulus, ultimate tensile stress, toughness, and elongation at break. Data are presented as mean ± standard deviation of three independent experiments. The two networks showed negligible differences in T_g and mechanical performance — including ultimate tensile stress, Young's modulus, toughness, and elongation at break, indicating that the trace HCO_3^- impurity present in the CO_2-derived TBAC has a minimal effect on the thermomechanical properties of the polymer networks.

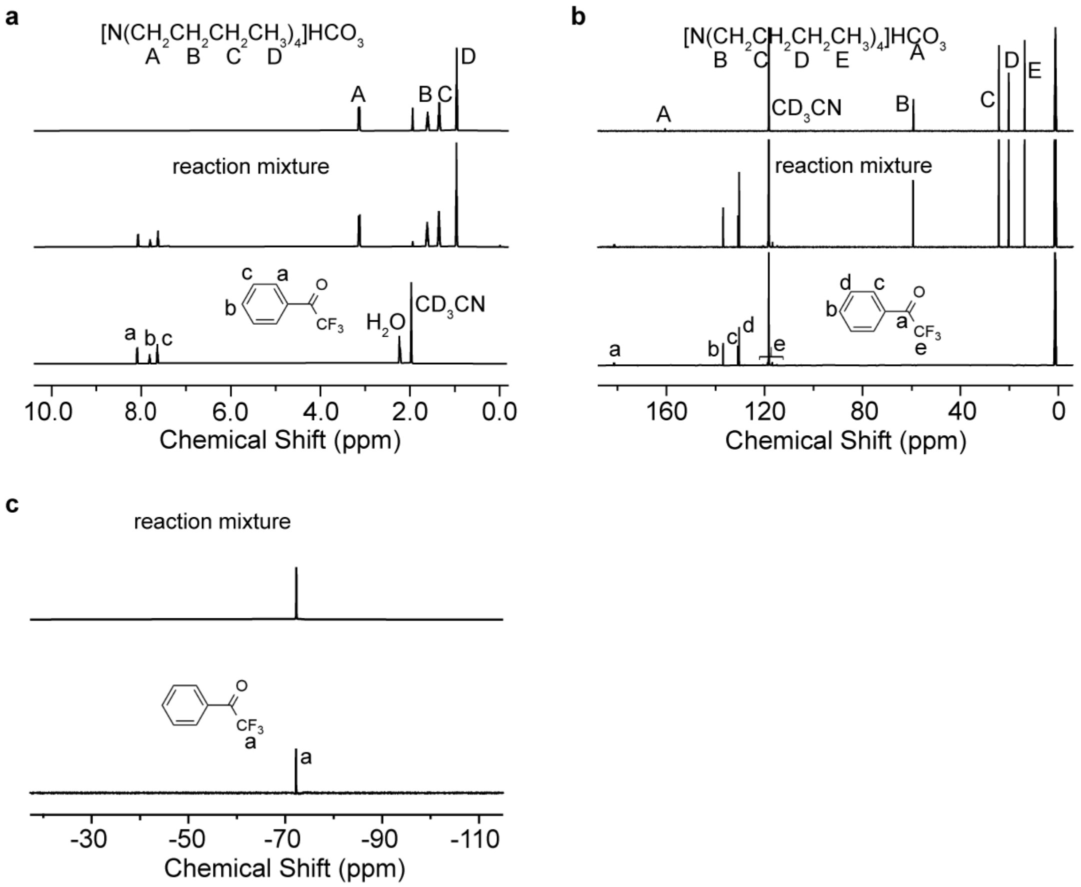

Supplementary Fig. 28 | NMR analysis of the reaction between TFP and HCO_3^-. a, ^1H NMR, **b**, ^{13}C NMR, and **c**, ^{19}F NMR spectra (in CD_3CN) for pure TFP, TBAHC (HCO_3^- source), and their reaction mixture. The spectra of the mixture are identical to those of the starting materials, indicating no detectable reaction. In sharp contrast to the quantitative reaction of TFP with CO_3^{2-} under identical conditions, this result confirms the high selectivity for CO_3^{2-} over HCO_3^-, which is likely attributable to the superior nucleophilicity of CO_3^{2-}. This indicates that the trace HCO_3^- impurity present in the CO_2-derived TBAC has a minimal effect on the cross-linking reaction.

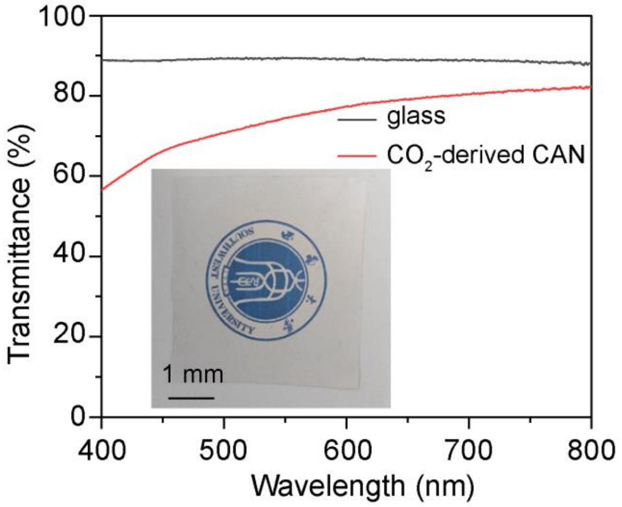

Supplementary Fig. 29 | UV-Vis transmission spectra of the CO_2-derived CANs films with thickness of ca. 0.5 mm. Inset is the photo of CANs film.

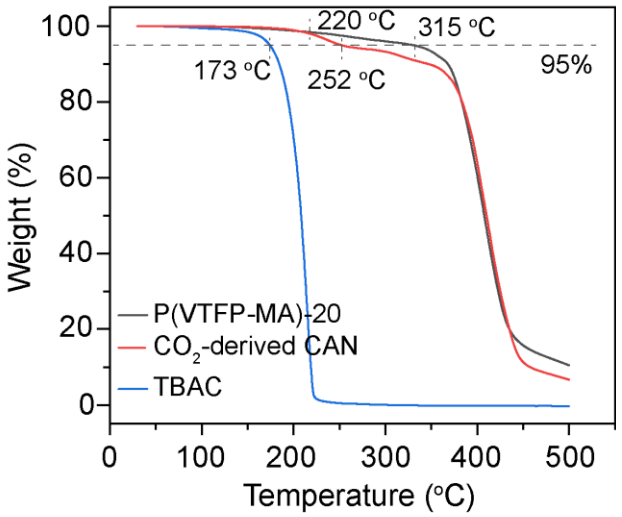

Supplementary Fig. 30 | TGA curves of CO_2-derived CAN, P(VTFP-MA)-20 and TBAC under air atmosphere. The results indicate that the decomposition temperatures at 5% mass loss ($T_{d,5\%}$) are 252 °C for the CO_2-derived CAN, 173 °C for TBAC, and 315 °C for P(VTFP-MA)-20, respectively. Although the CO_2-derived CAN exhibited reduced thermal stability relative to linear polymer P(VTFP-MA)-20, their negligible mass loss at 220°C confirms effective stabilization of the thermally labile TBAC component through network formation.

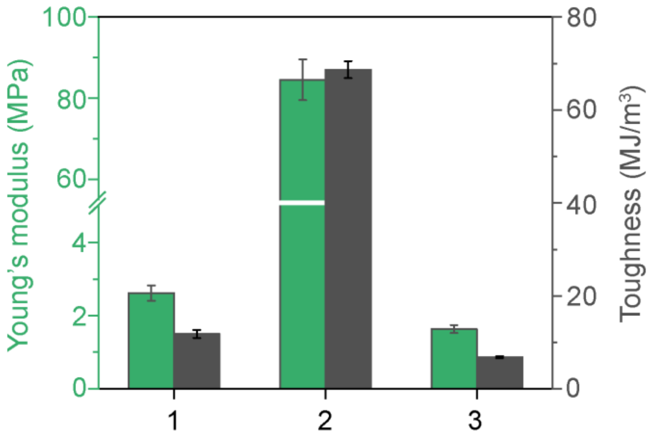

Supplementary Fig. 31 | Young's modulus and toughness of samples 1-3 as follows: 1, P(VTFP-MA)-20; 2, CO_2-derived CAN; 3, P(VTFP-MA)-20 with TBAI additive. Data are presented as mean ± standard deviation of three independent experiments.

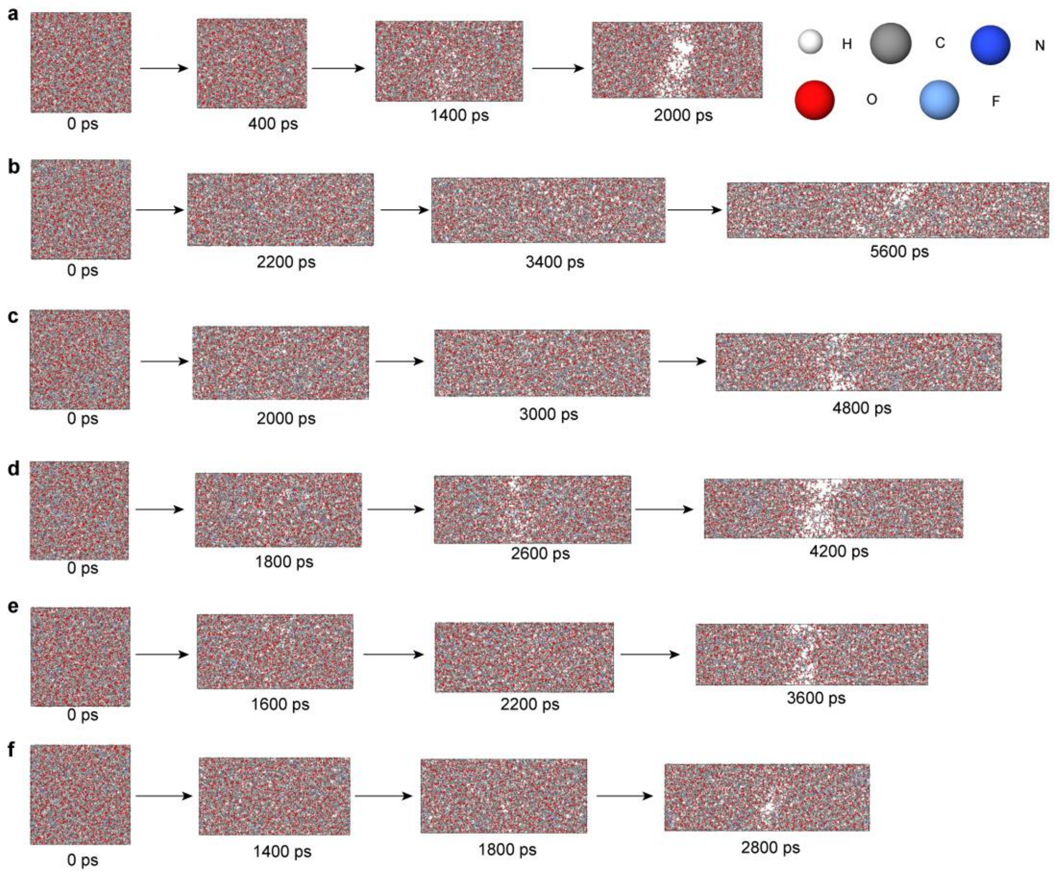

Supplementary Fig. 32 | Structural evolution of CANs during tensile deformation from MD simulations. Representative snapshots show the deformation process under uniaxial stretching for: (**a**) the control system (H^+), (**b**) CANs containing TBA^+, (**c**) CAN containing TPA^+, (**d**) CAN containing TEA^+, (**e**) CAN containing TMA^+, and (**f**) CAN containing $BnMA^+$ as counter-cations.

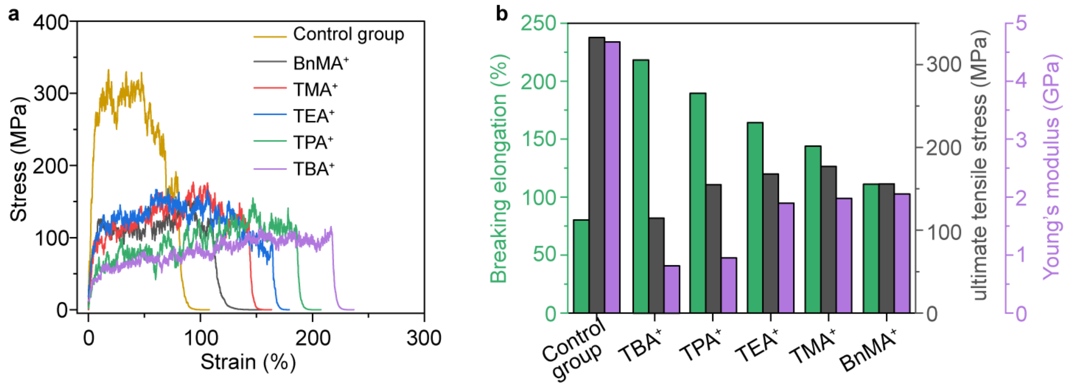

Supplementary Fig. 33 | Simulated stress-strain curves (a), and the corresponding elongation at break, ultimate tensile stress and Young's modulus (b).

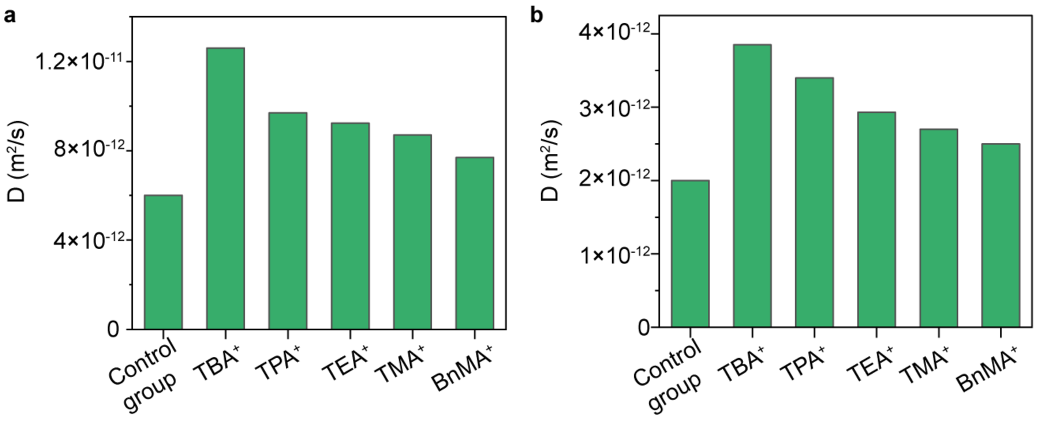

Supplementary Fig. 34 | Diffusion coefficients of the CANs. Comparison of the diffusion coefficients for (a) the counter-cations and (b) the anionic polymer chain segments, as calculated from their MSD.

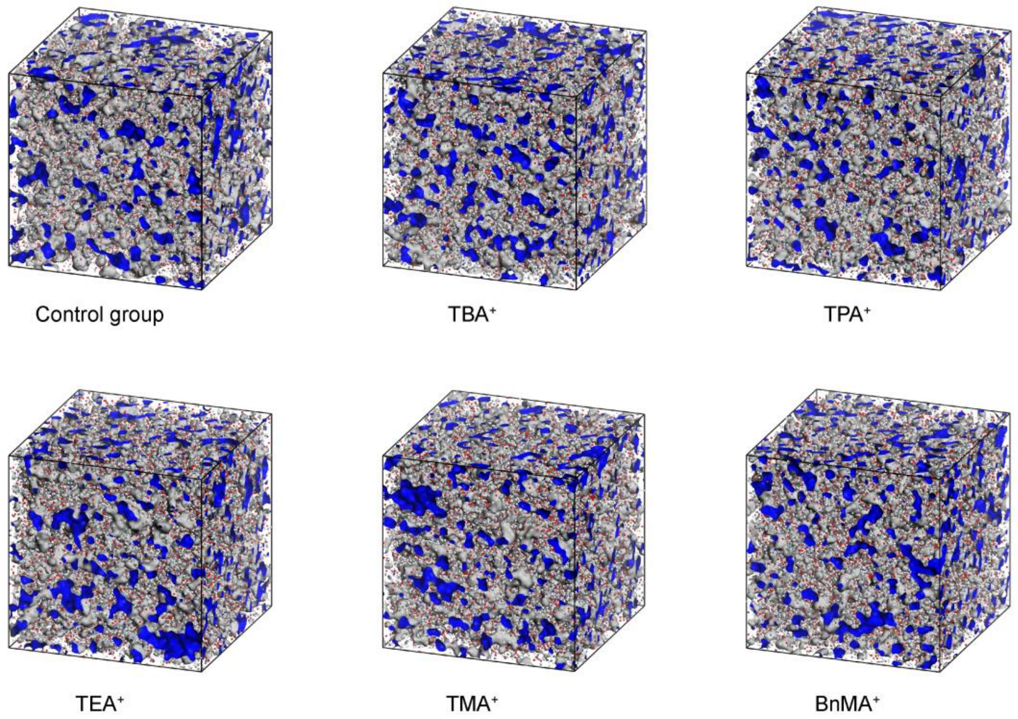

Supplementary Fig. 35 | FFV of CANs.

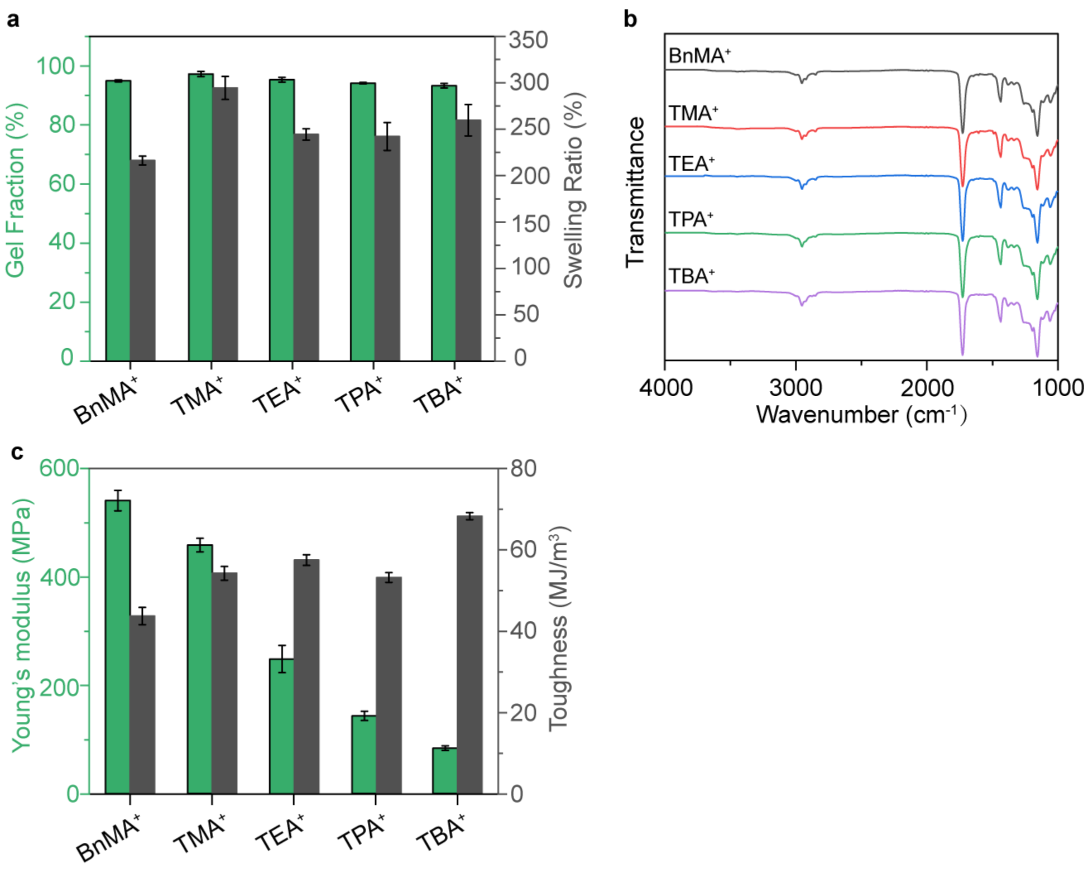

Supplementary Fig. 36 | Influence of counter-cations on the properties of CANs. a, Gel fraction and swelling ratio. **b,** FTIR spectra. **c,** Young's modulus and toughness. Data are presented as mean ± standard deviation of three independent experiments.

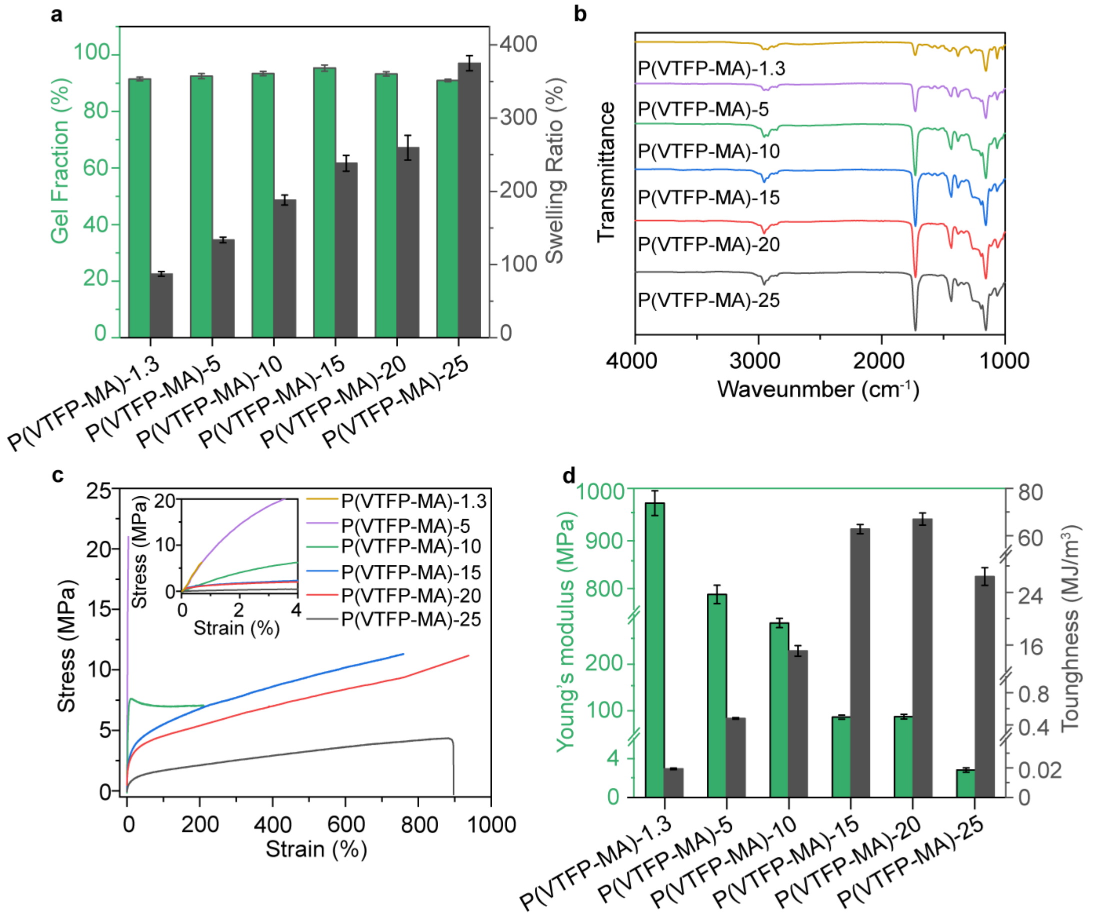

Supplementary Fig. 37 | Influence of the MA-to-VTFP molar ratio on the properties of the CANs. a, Gel fraction and swelling ratio. **b**, FTIR spectra. **c**, Tensile stress-strain curves. **d**, Young's moduli and toughness derived from the curves in (c). Data are presented as mean ± standard deviation of three independent experiments.

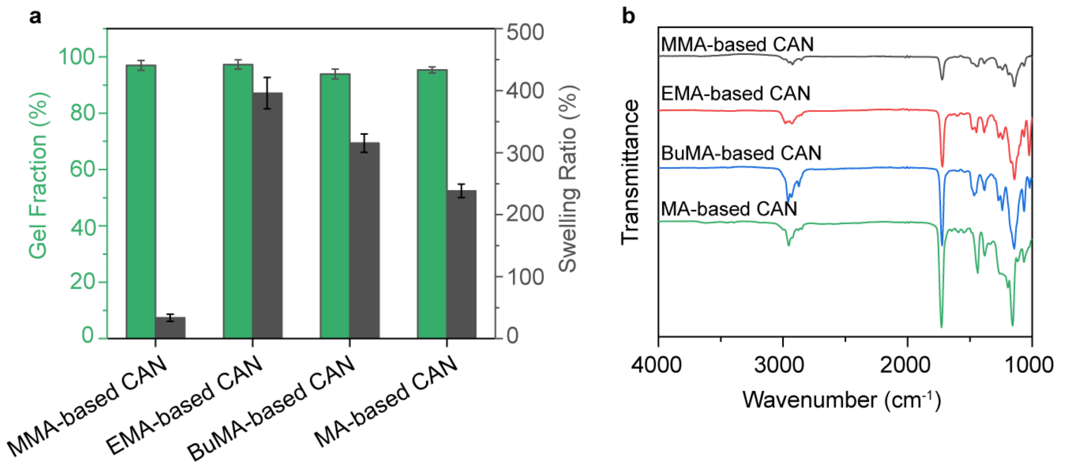

Supplementary Fig. 38 | Influence of comonomers on the properties of the CANs. **a**, Gel fraction and swelling ratio. **b**, FTIR spectra. Data are presented as mean ± standard deviation of three independent experiments.

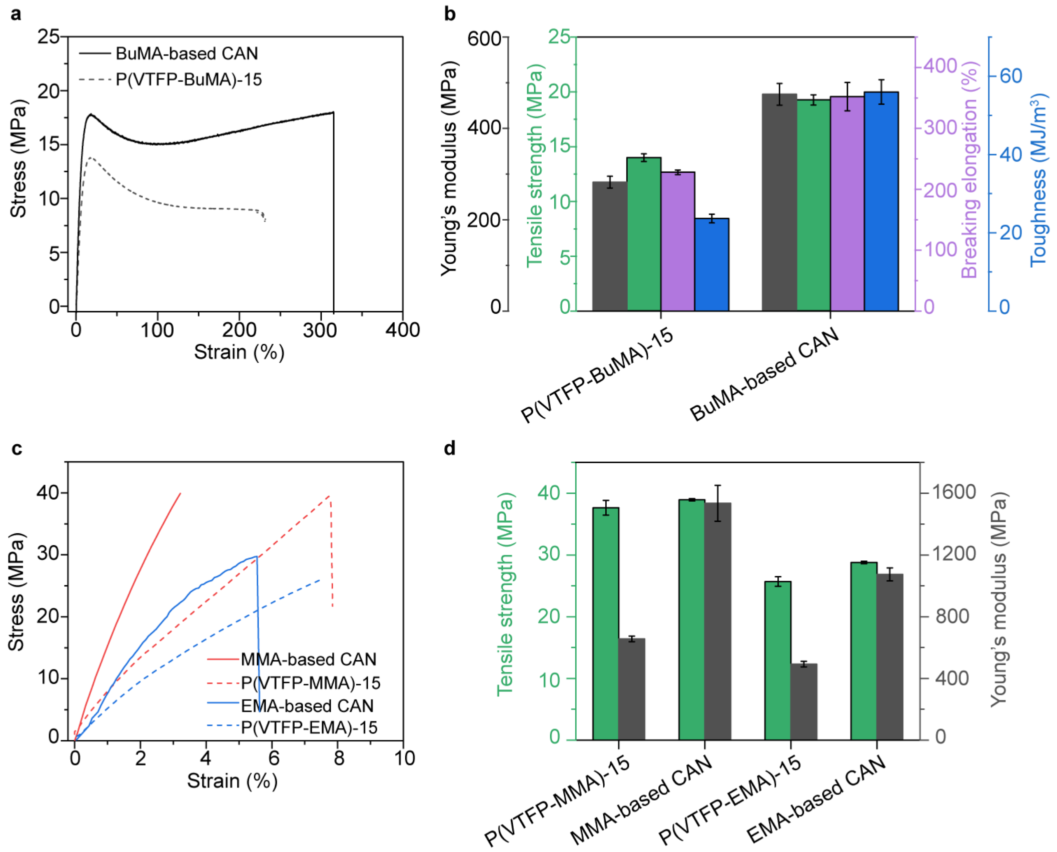

Supplementary Fig. 39 | Mechanical properties of linear precursor polymers and their corresponding CANs. a, Tensile stress-strain curves of P(VTFP-BuMA)-15 and its corresponding CAN. **b,** Comparison of ultimate tensile stress, Young's modulus, toughness, and elongation at break for the samples in (a). **c**, Tensile stress-strain curves of P(VTFP-MMA)-15 and P(VTFP-EMA)-15, alongside their corresponding CANs. **d**, Comparison of Young's modulus and toughness for the samples in (c). Data are presented as mean ± standard deviation of three independent experiments.

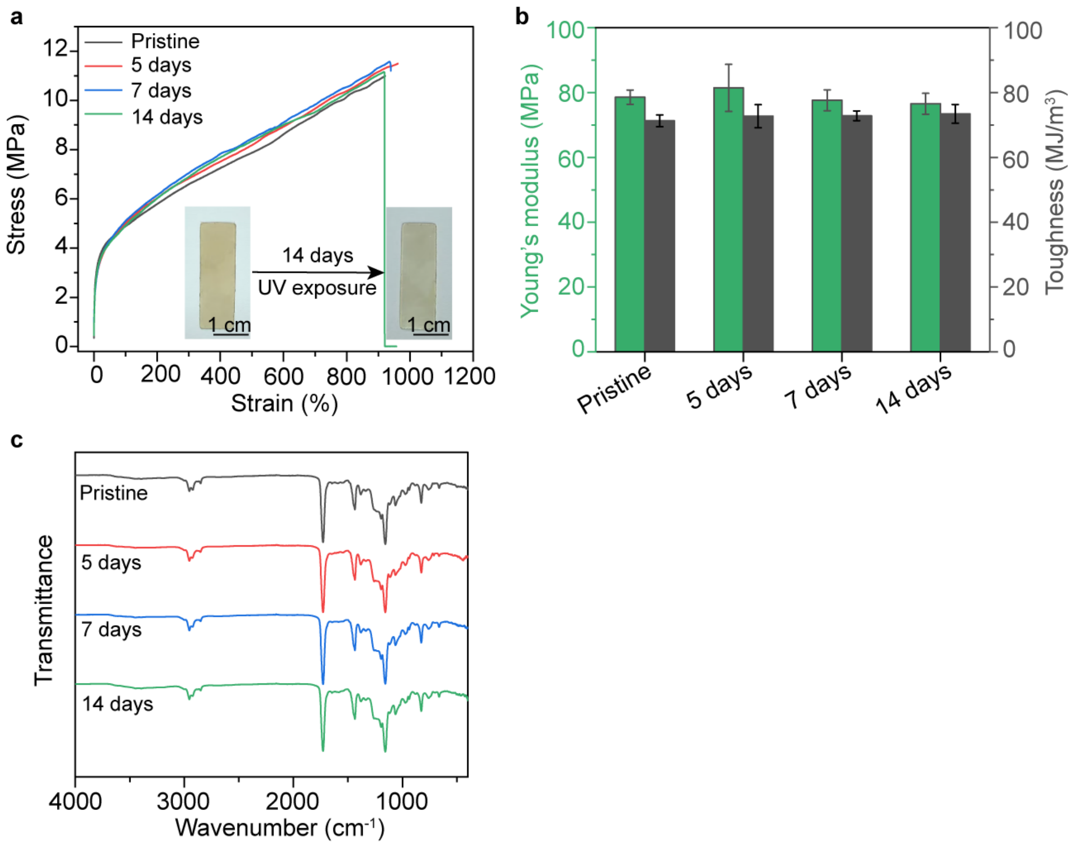

Supplementary Fig. 40 | UV stability of the CO_2-derived CAN. a, Stress-strain curves before and after various UV exposure durations. *Inset:* Representative photographs of a sample before and after 14 days of UV exposure. **b,** Young's modulus and toughness from (a). **c,** FTIR spectra before and after various UV exposure durations. Data are presented as mean ± standard deviation of three independent experiments.

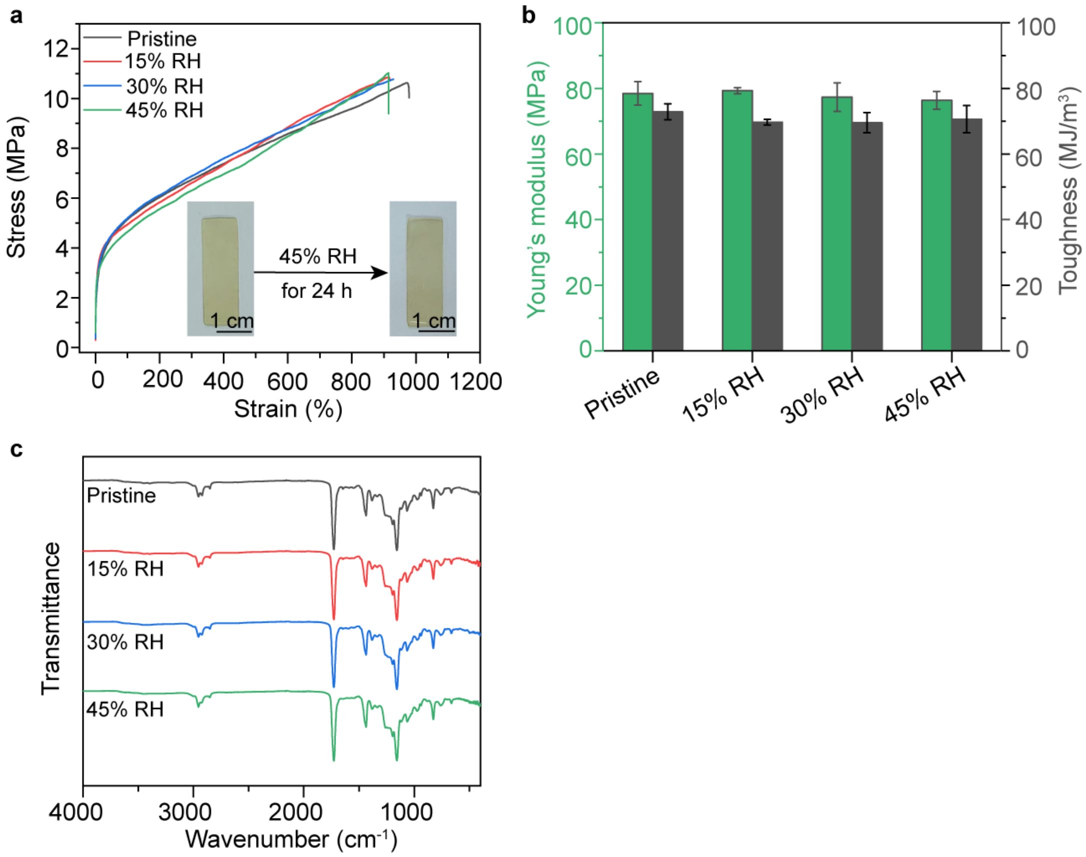

Supplementary Fig. 41 | Humidity stability of the CO_2-derived CAN. a, Stress-strain curves before and after conditioning at various relative humidity **(**RH) levels for 24 hours. *Inset:* Representative photographs of a sample before and after 24 hours of 45% RH exposure. **b,** Young's modulus and toughness from (a). **c,** FTIR spectra before and after conditioning at various RH levels for 24 hours. Data are presented as mean ± standard deviation of three independent experiments.

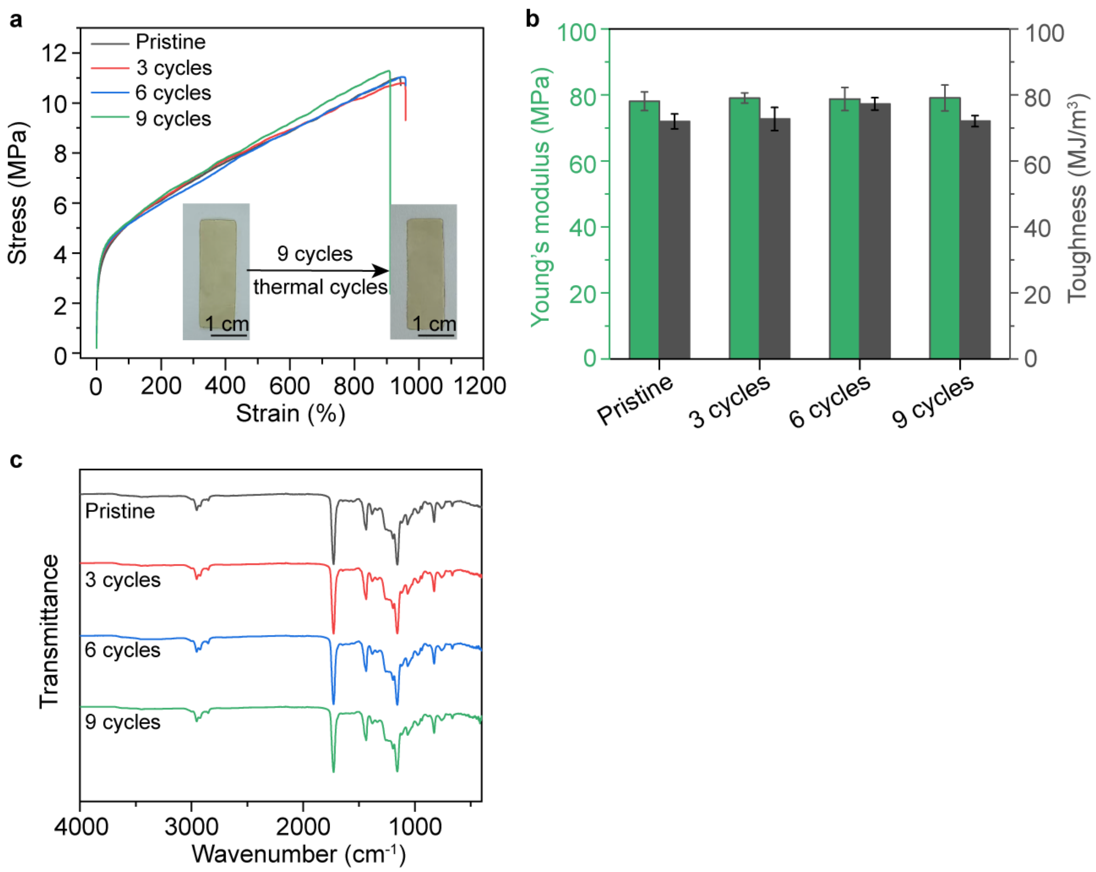

Supplementary Fig. 42 | Thermal cycling stability of the CO_2-derived CAN. a, Stress-strain curves before and after various thermal cycles. *Inset:* Representative photographs of a sample before and after 9 thermal cycles. **b,** Young's modulus and toughness from (a). **c,** FTIR spectra before and after various thermal cycles. Data are presented as mean ± standard deviation of three independent experiments.

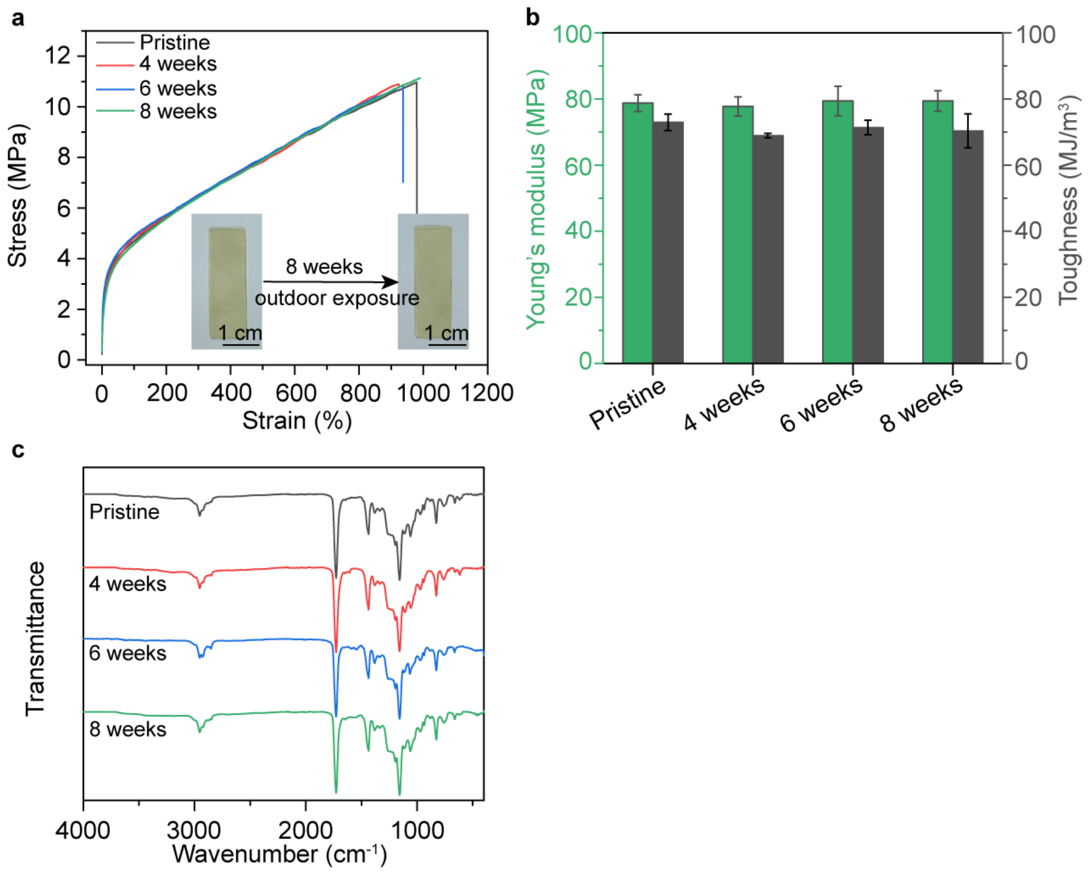

Supplementary Fig. 43 | Outdoor stability of the CO_2-derived CAN. a, Stress-strain curves of samples after outdoor exposure for various durations. *Inset:* Representative photographs of a sample before and after 8 weeks of outdoor exposure. **b,** Young's modulus and toughness derived from (a). **c,** FTIR spectra of the CO_2-derived CAN recorded after outdoor exposure for various durations. Data are presented as mean ± standard deviation of three independent experiments.

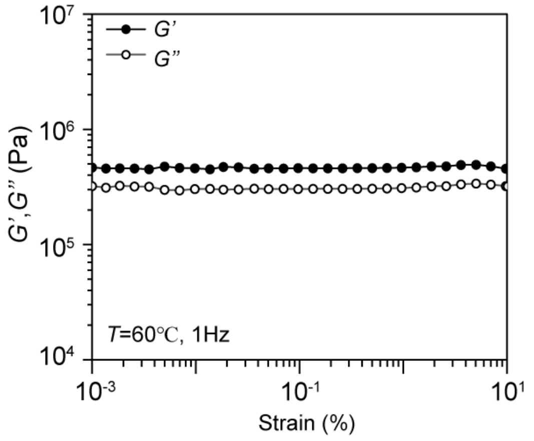

Supplementary Fig. 44 | A strain sweep test conducted at 60 °C. The linear viscoelastic regime was identified within a strain amplitude range of γ = 0.001%-10%, where the *G'* remained constant.

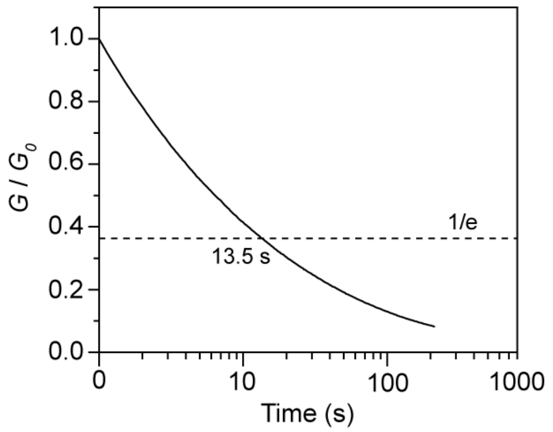

Supplementary Fig. 45 | Stress-relaxation curve at 60 °C. The relaxation time (τ) was 13.5 seconds.

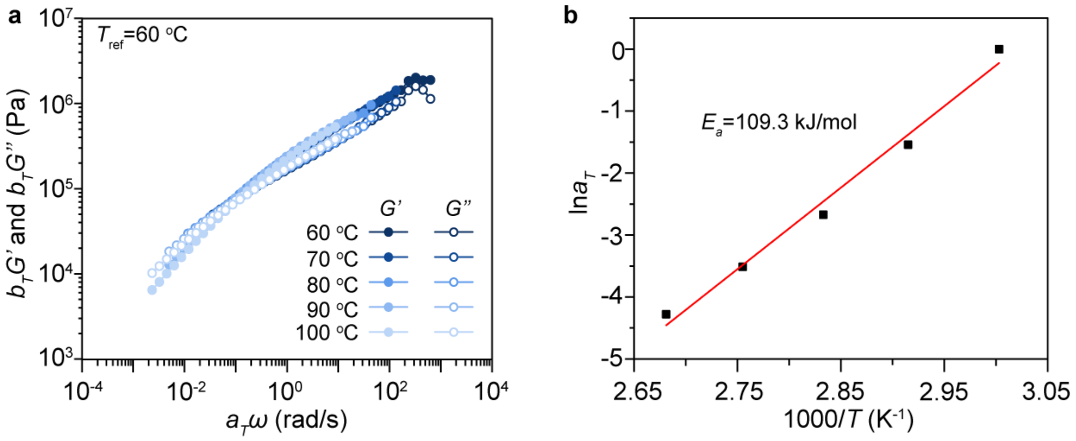

Supplementary Fig. 46 | Master curve of CO_2-derived CAN based on TTS of SAOS experiments at a reference temperature of 60 °C (a), and the corresponding Arrhenius plot of horizontal shift factors (a_T) versus 1000/T (b).

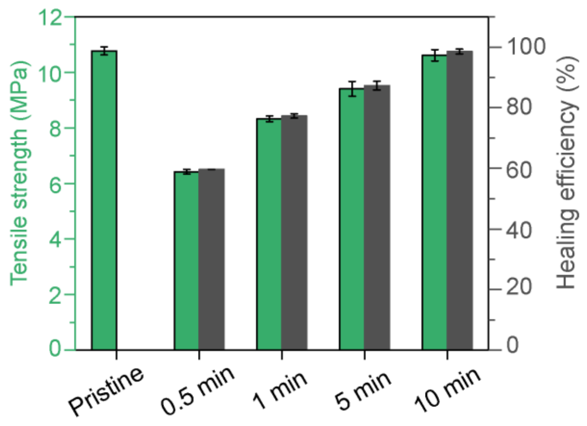

Supplementary Fig. 47 | Ultimate tensile stress and healing efficiency of repaired samples after different healing durations under 0.5 MPa pressure at 80°C. Data are presented as mean ± standard deviation of three independent experiments.

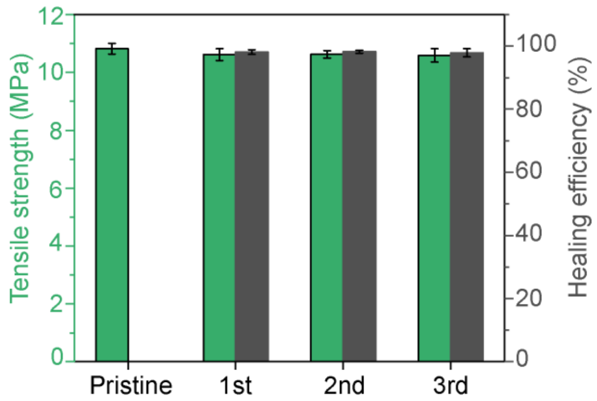

Supplementary Fig. 48 | Ultimate tensile stress and healing efficiency after three damage-healing cycles. Data are presented as mean ± standard deviation of three independent experiments.

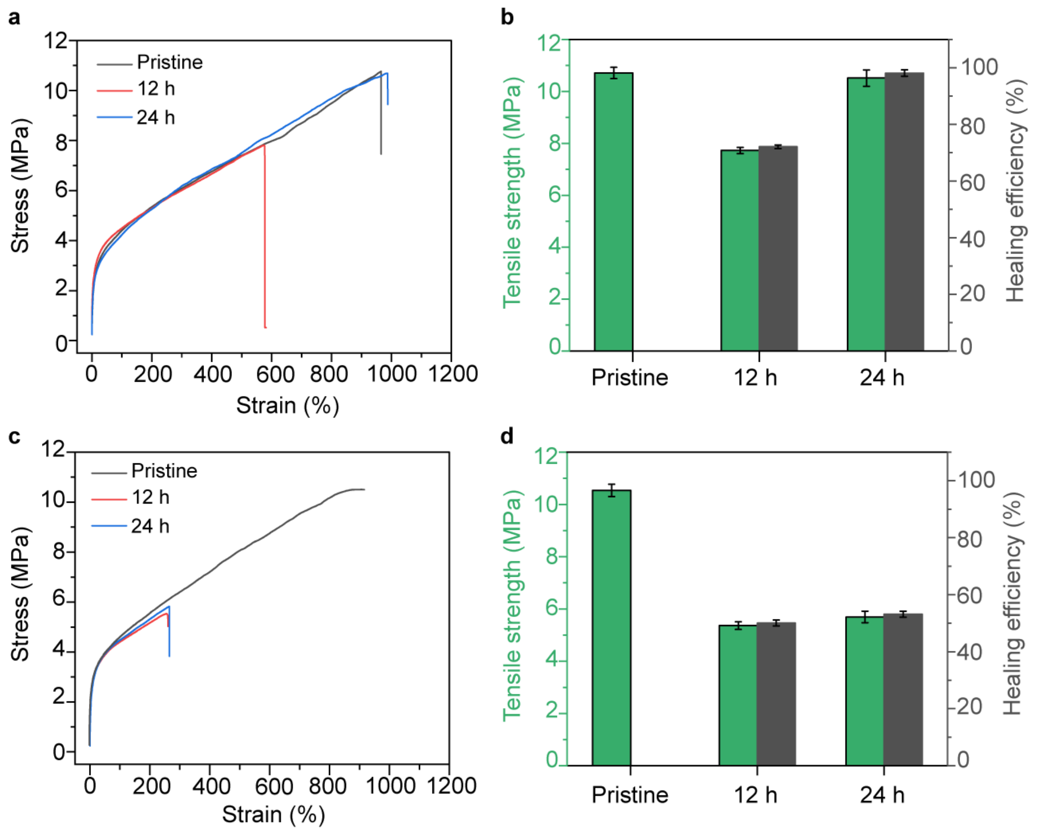

Supplementary Fig. 49 | Low-temperature self-healing performance. a-b, Stress–strain curves of the repaired samples after different healing durations under 2 MPa at 40 °C (**a**), and the corresponding ultimate tensile stress and healing efficiencies (**b**). **c-d**, Stress–strain curves of the repaired samples after different healing durations under 2 MPa at room temperature (c), and the corresponding ultimate tensile stress and healing efficiencies (**d**). Data are presented as mean ± standard deviation of three independent experiments.

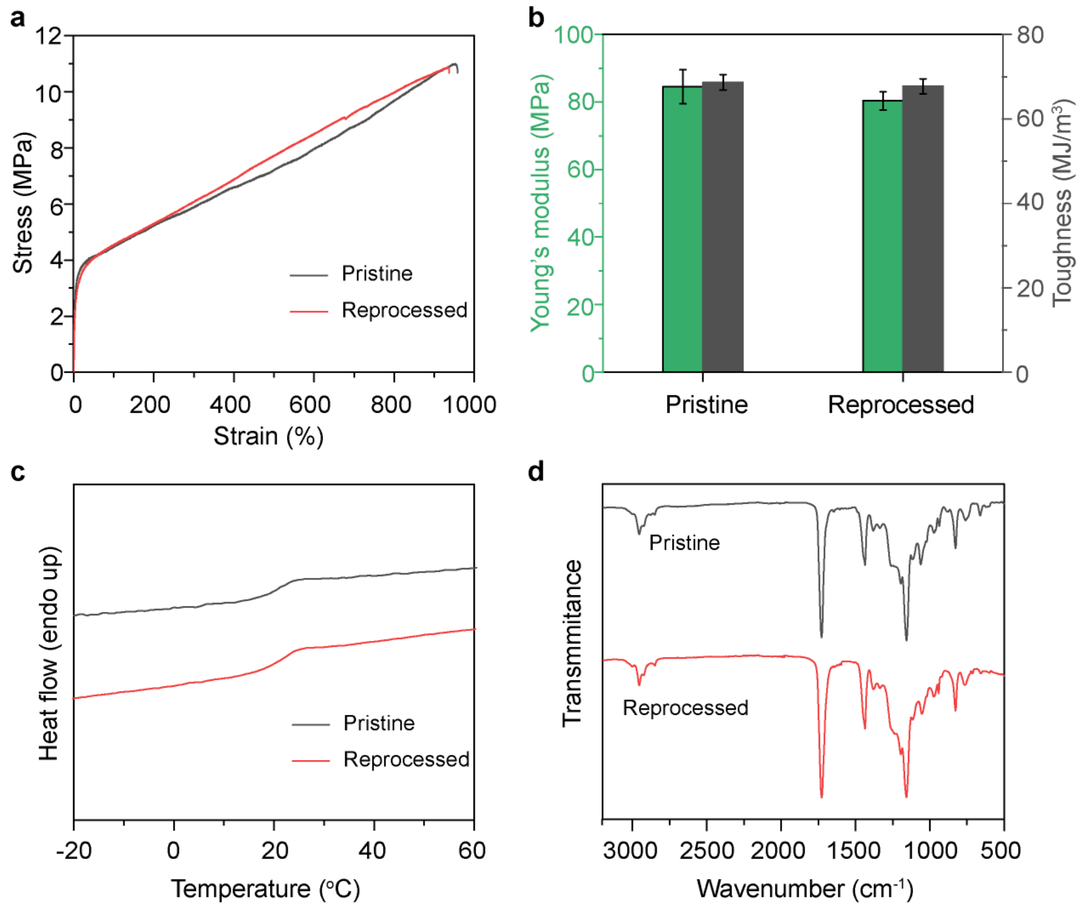

Supplementary Fig. 50 | Comparison of mechanical properties (a-b), thermal characteristics (c), and chemical composition (d) before and after injection molding. Data are presented as mean ± standard deviation of three independent experiments.

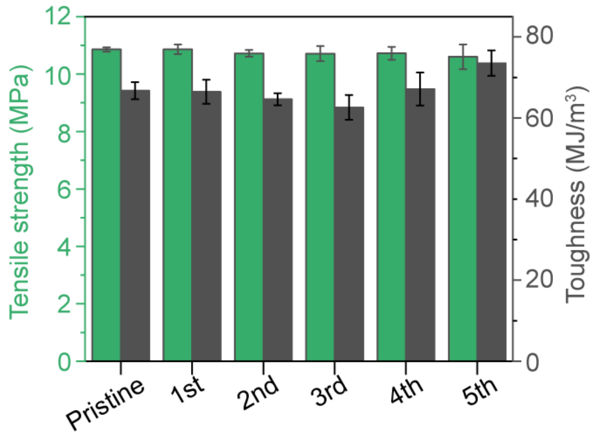

Supplementary Fig. 51 | Ultimate tensile stress and toughness of the original and reprocessed CANs samples after multiple hot-pressing cycles. Data are presented as mean ± standard deviation of three independent experiments.

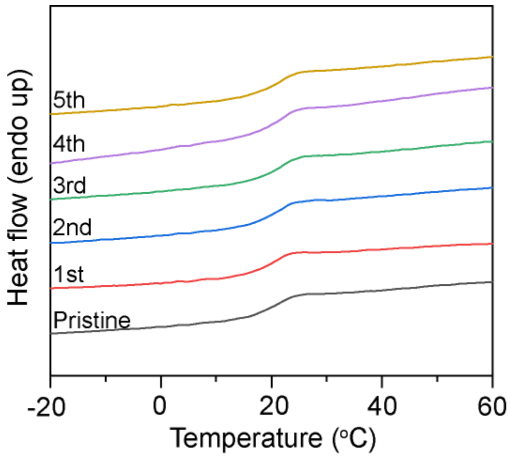

Supplementary Fig. 52 | DSC curves of pristine and reprocessed CAN after multiple hot-pressing cycles.

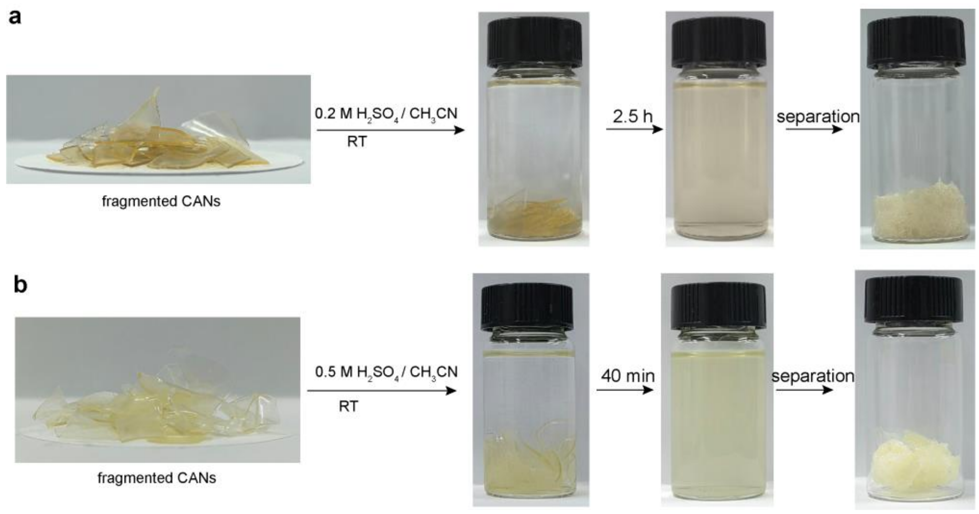

Supplementary Fig. 53 | Photographs showing chemical recycling of fragmented CANs in CH_3CN solution of (a) 0.2 M H_2SO_4 and (b) 0.5 M H_2SO_4.

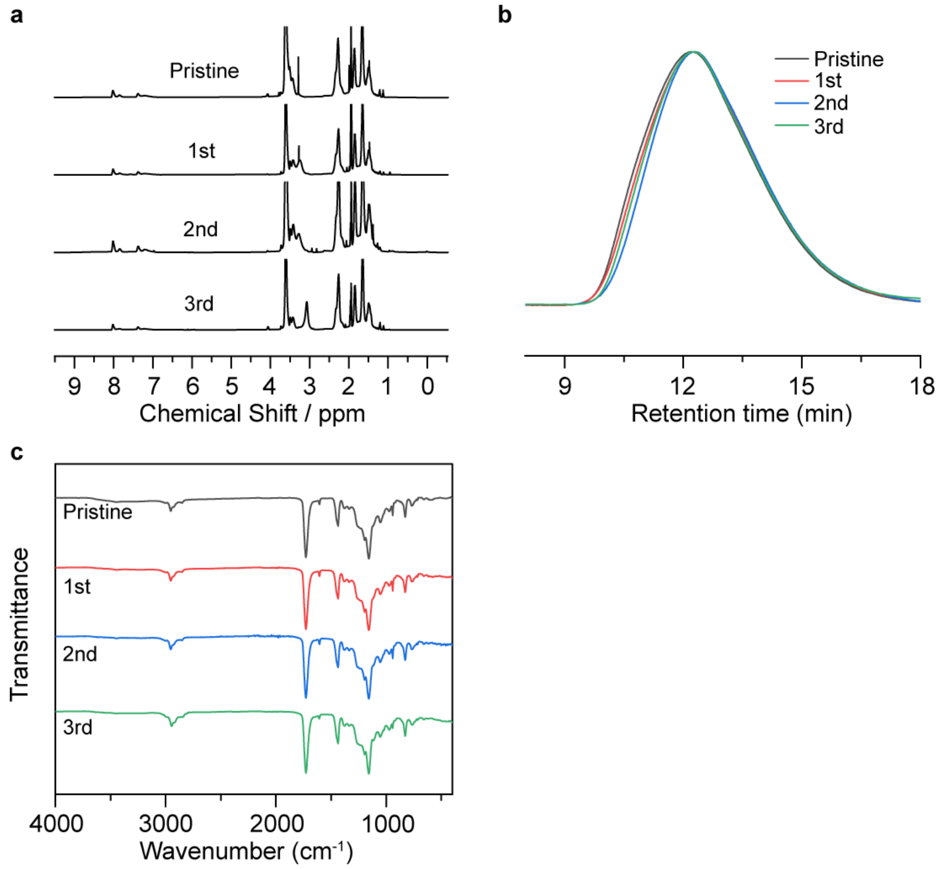

Supplementary Fig. 54 | (a) ^{1}H NMR spectrum (in CD_3CN), (b) GPC spectrum (using THF as the eluent) and (c) IR spectrum of the original and recycled P(VTFP-MA)-20.

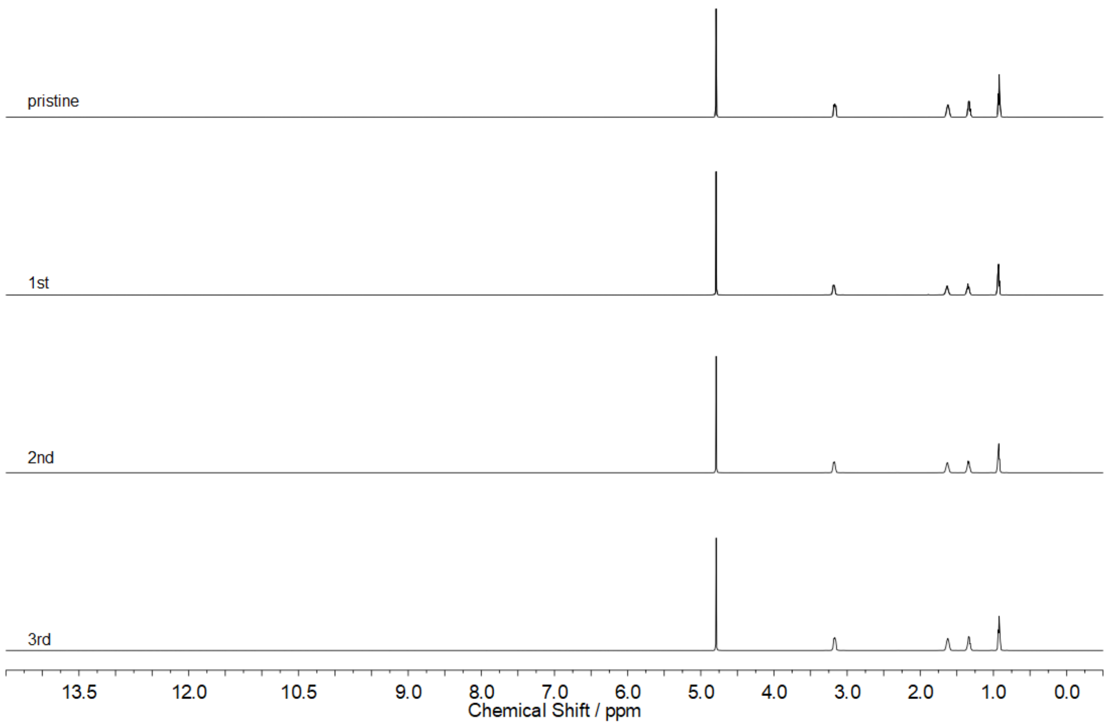

Supplementary Fig. 55 | ^{1}H NMR spectrum (in D_2O) of the original and recycled TBAH.

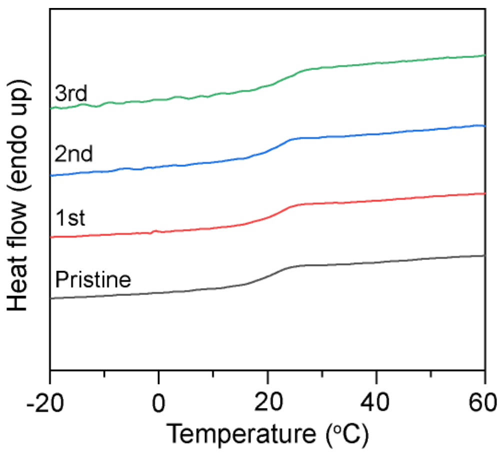

Supplementary Fig. 56 | DSC curves of the original and reborn CAN.

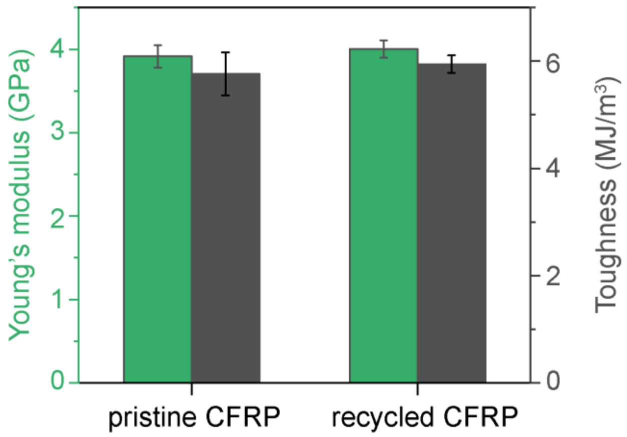

Supplementary Fig. 57 | Young's modulus and toughness of original and reborn CANs-based CFRP. Data are presented as mean ± standard deviation of three independent experiments.

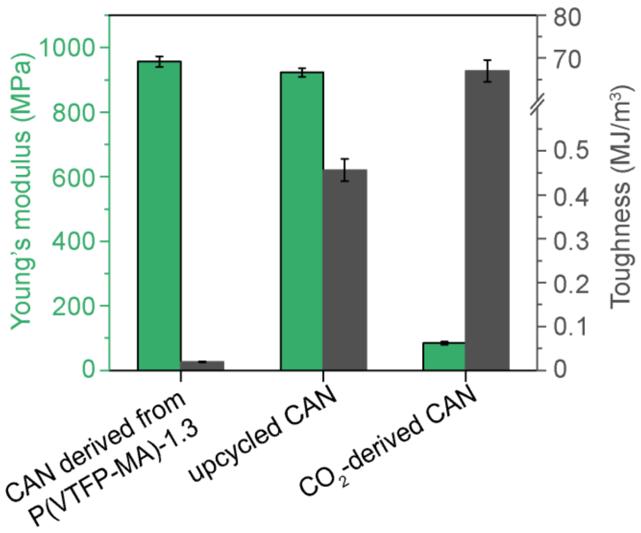

Supplementary Fig. 58 | Young's modulus and toughness of original and upcycled CAN. Data are presented as mean ± standard deviation of three independent experiments.

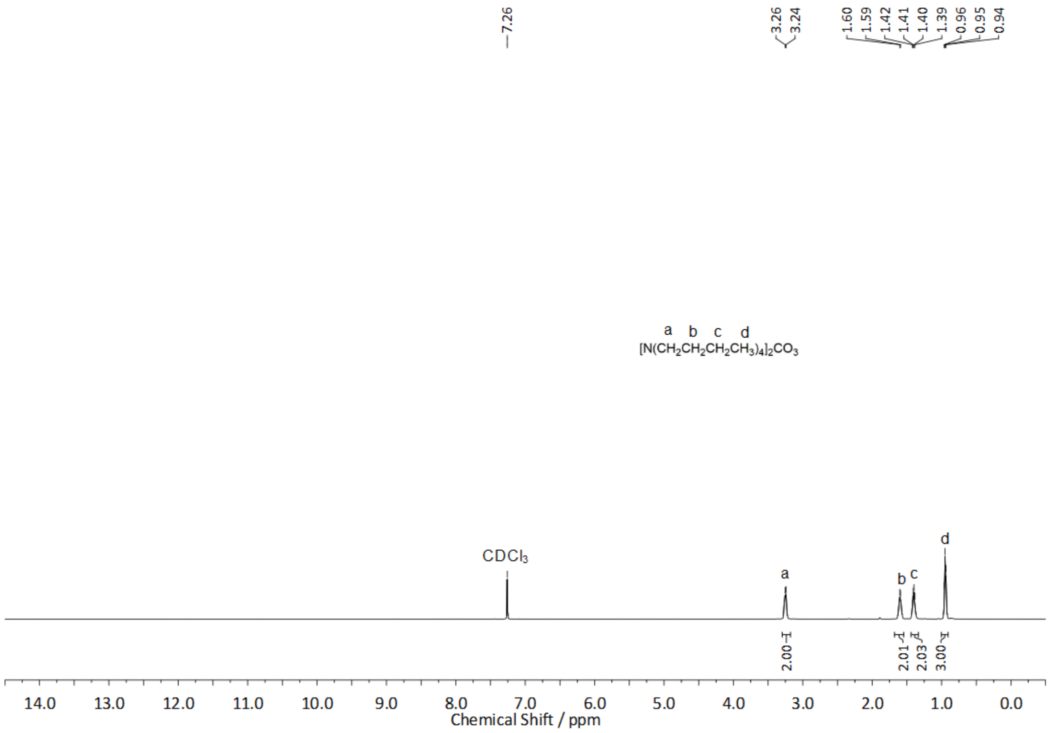

Supplementary Fig. 59 | ^{1}H NMR spectrum (in $CDCl_3$) of TBAC.

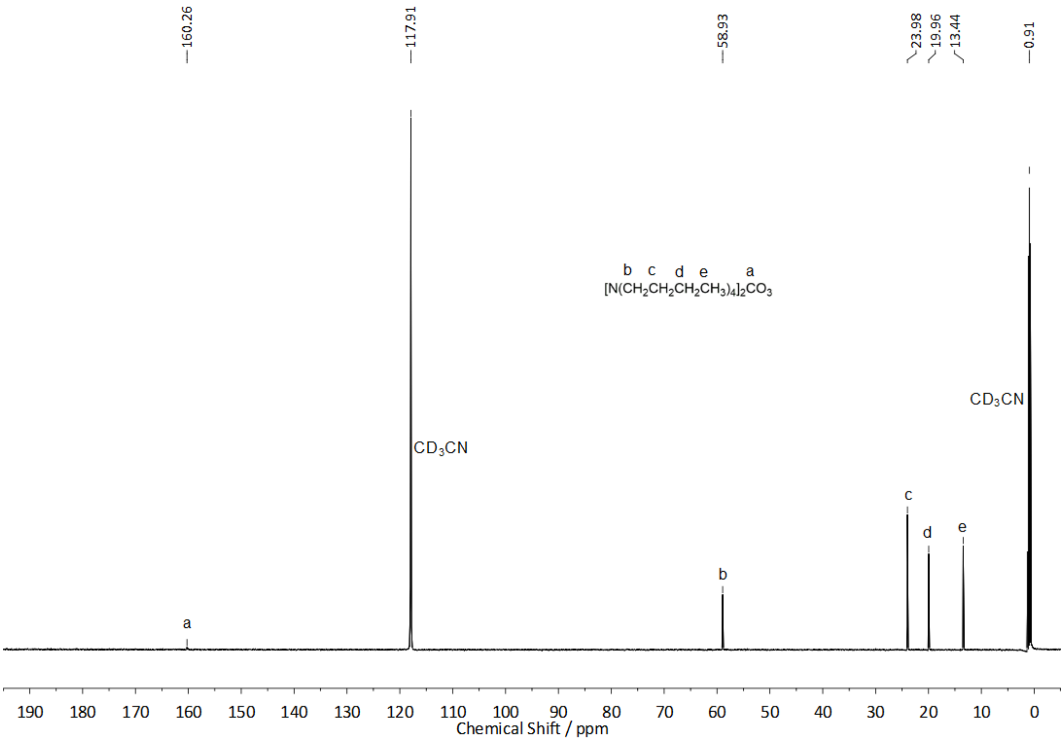

Supplementary Fig. 60 | ^{13}C NMR spectrum (in CD_3CN) of TBAC.

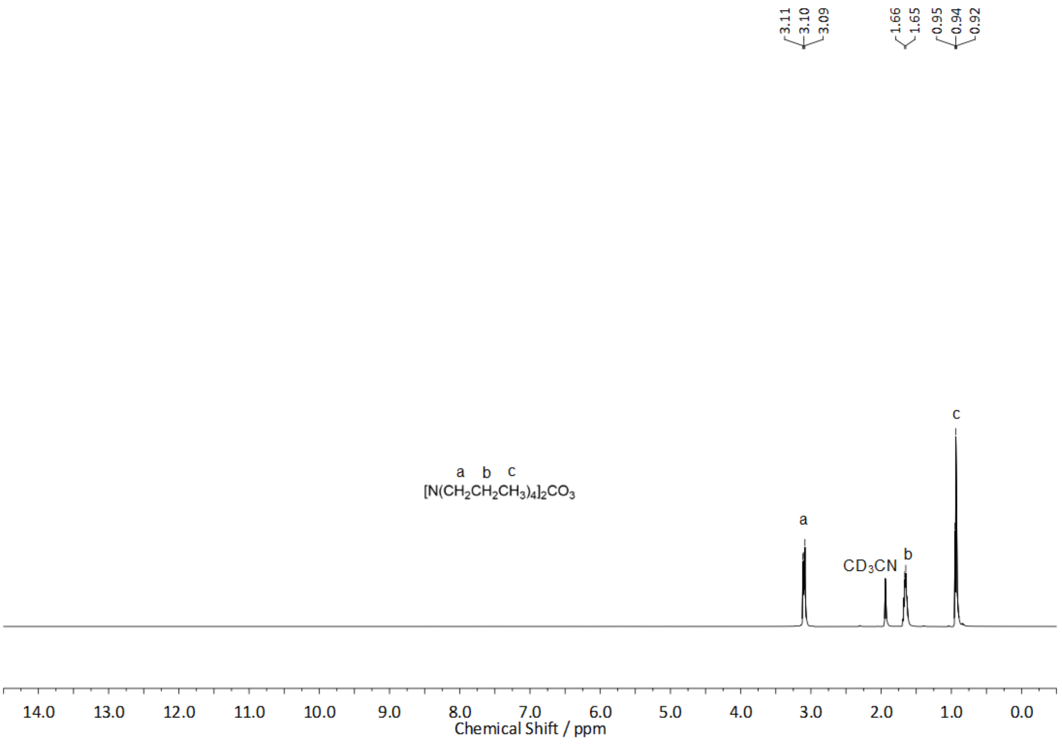

Supplementary Fig. 61 | ^{1}H NMR spectrum (in CD_3CN) of TPAC.

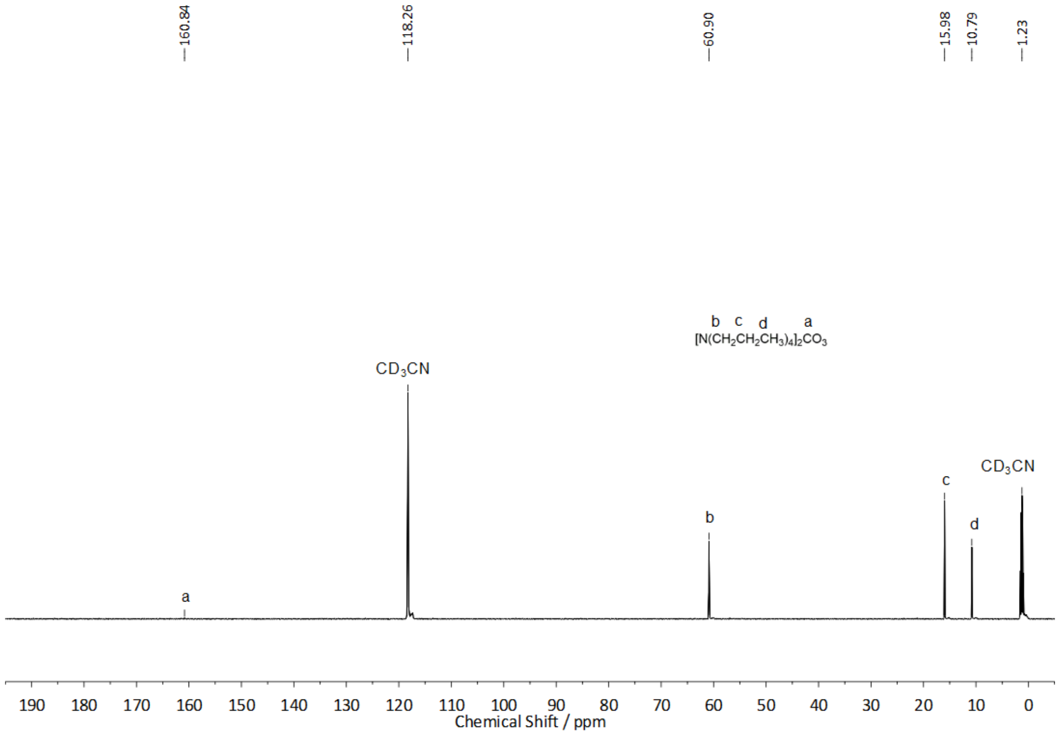

Supplementary Fig. 62 | ^{13}C NMR spectrum (in CD_3CN) of TPAC.

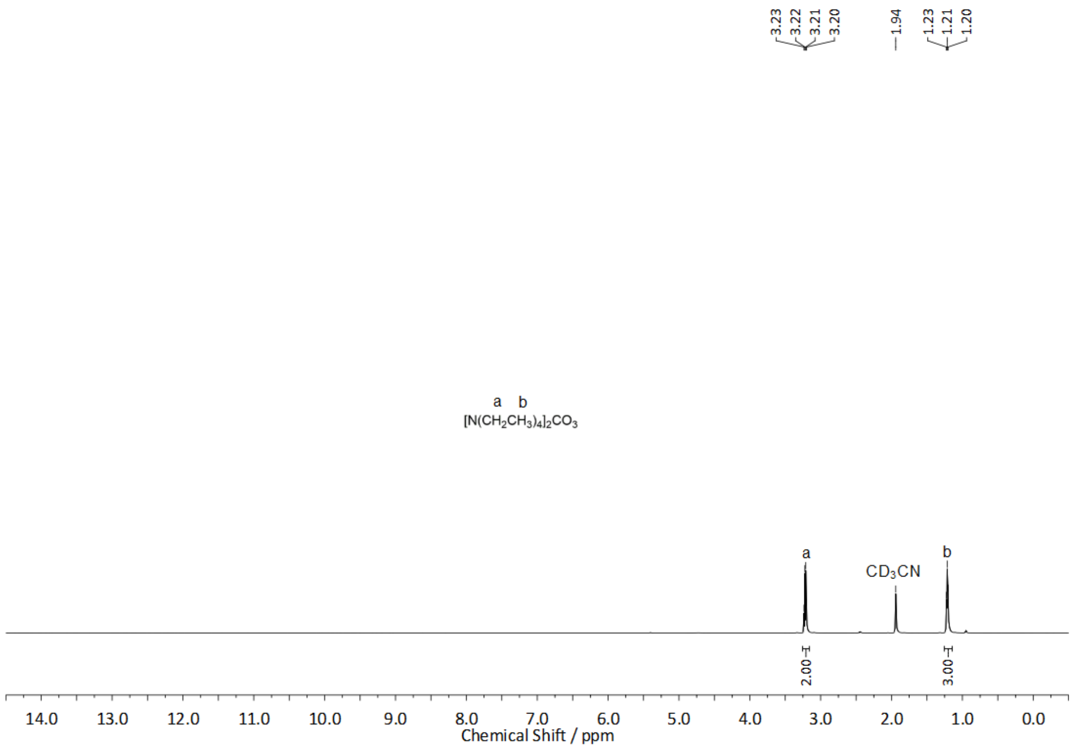

Supplementary Fig. 63 | ^{1}H NMR spectrum (in CD_3CN) of TEAC.

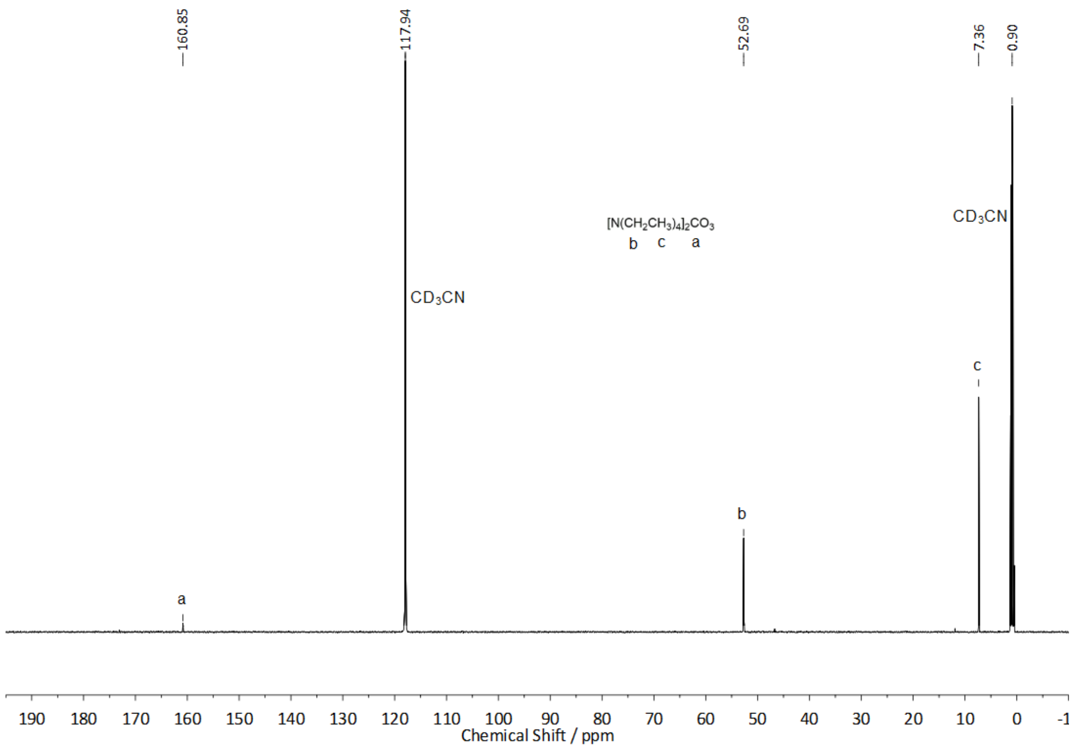

Supplementary Fig. 64 | ^{13}C NMR spectrum (in CD_3CN) of TEAC.

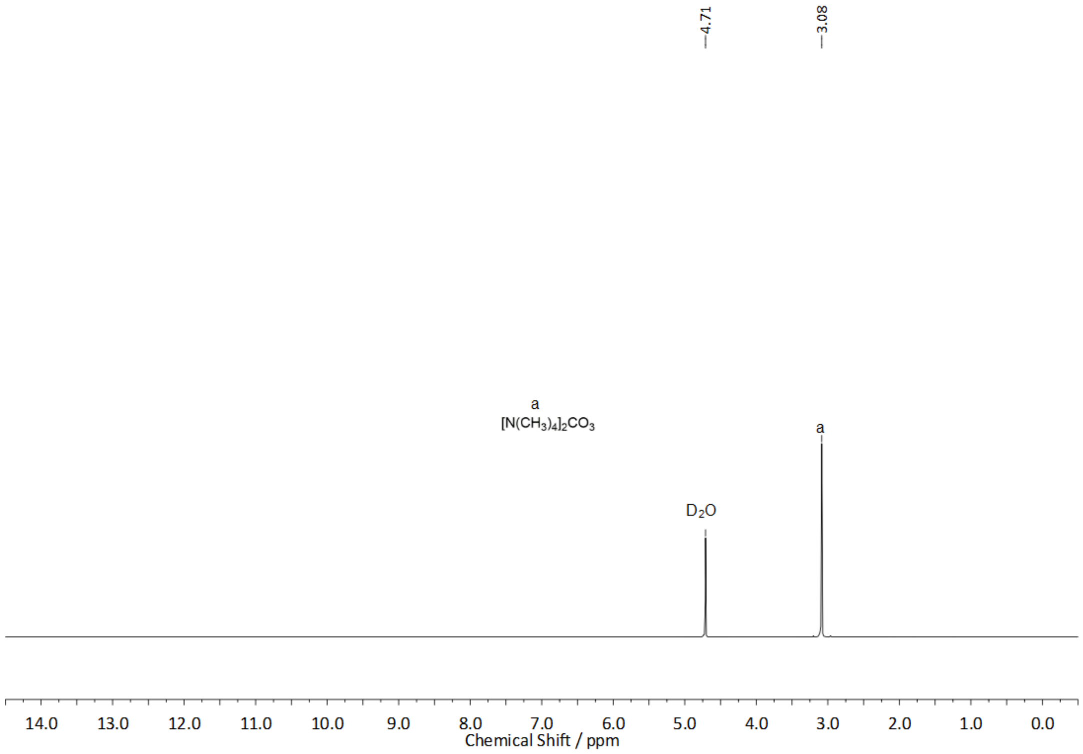

Supplementary Fig.65 | ^{1}H NMR spectrum (in D_2O) of TMAC.

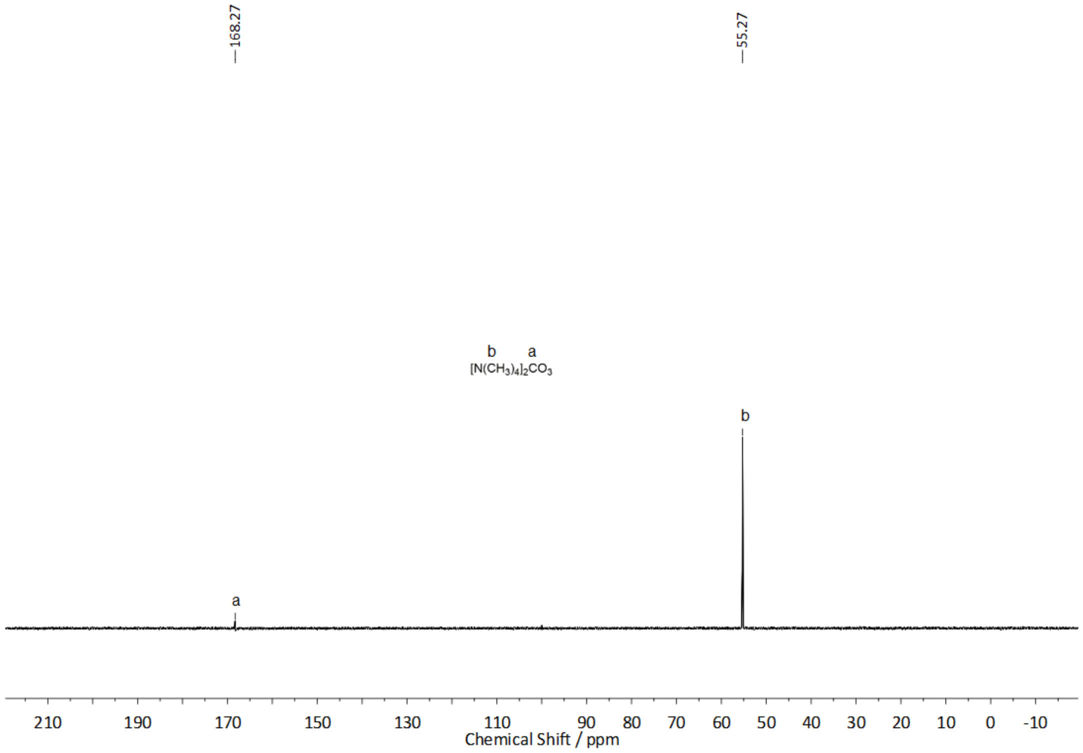

Supplementary Fig. 66 | ^{13}C NMR spectrum (in D_2O) of TMAC.

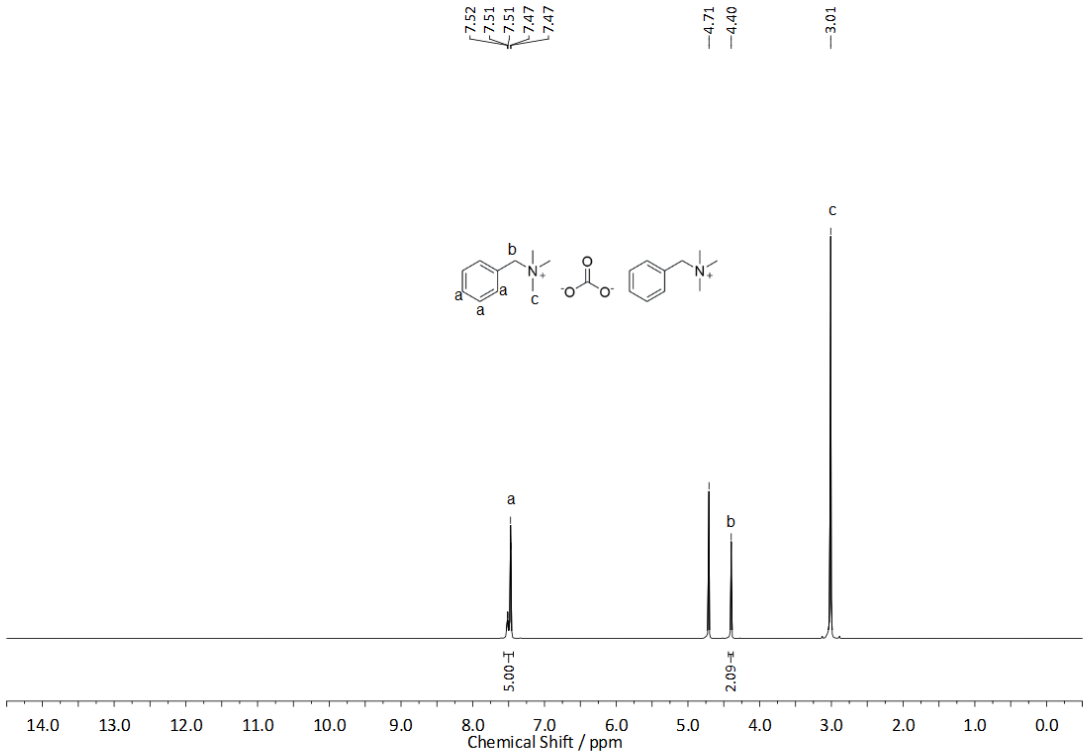

Supplementary Fig. 67 | ^{1}H NMR spectrum (in D_2O) of BnMAC.

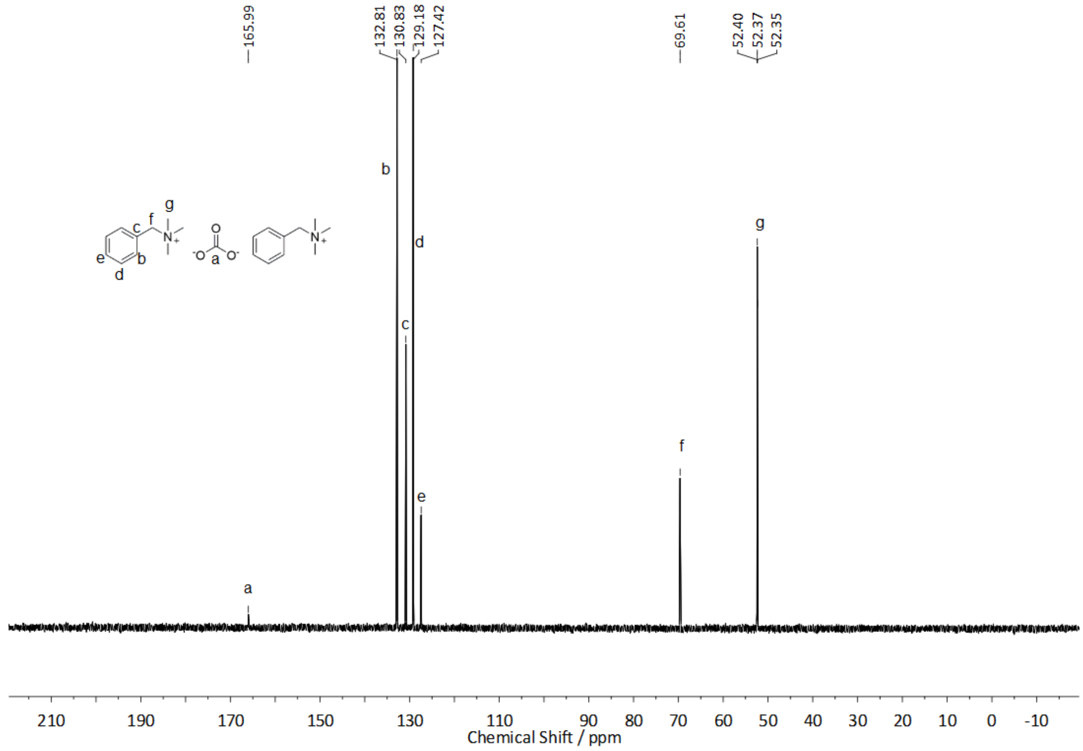

Supplementary Fig. 68 | ^{13}C NMR spectrum (in D_2O) of BnMAC.

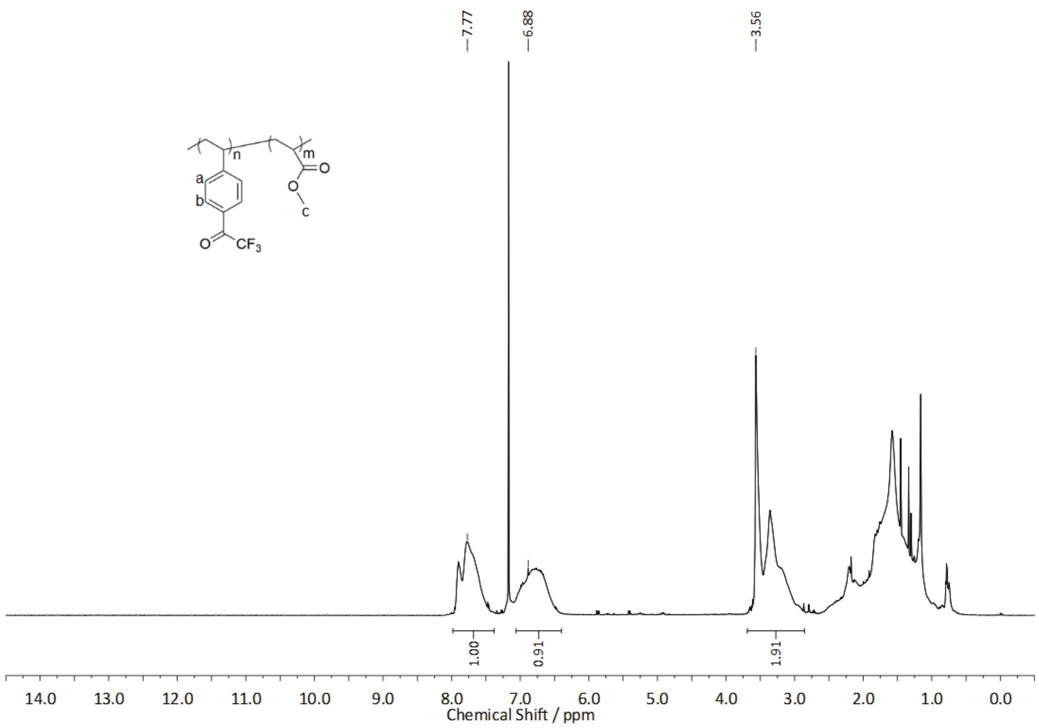

Supplementary Fig. 69 | ^{1}H NMR spectrum (in $CDCl_3$) of liner copolymer P(VTFP-MA)-1.3.

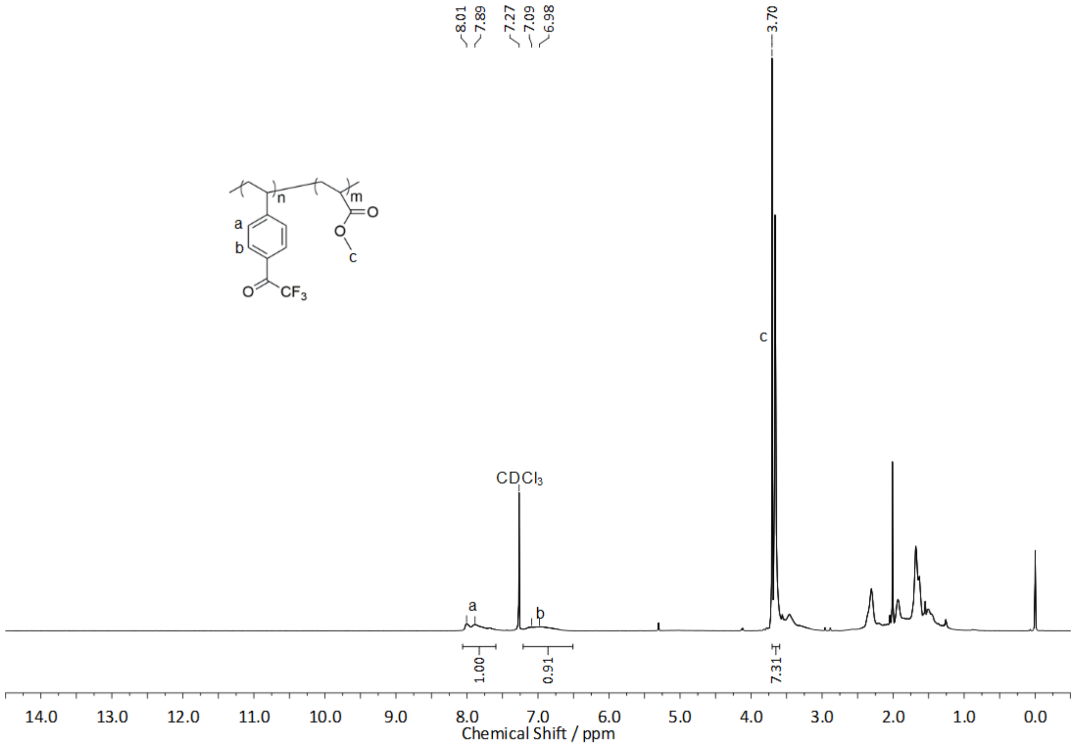

Supplementary Fig. 70 | ^{1}H NMR spectrum (in $CDCl_3$) of liner copolymer P(VTFP-MA)-5.

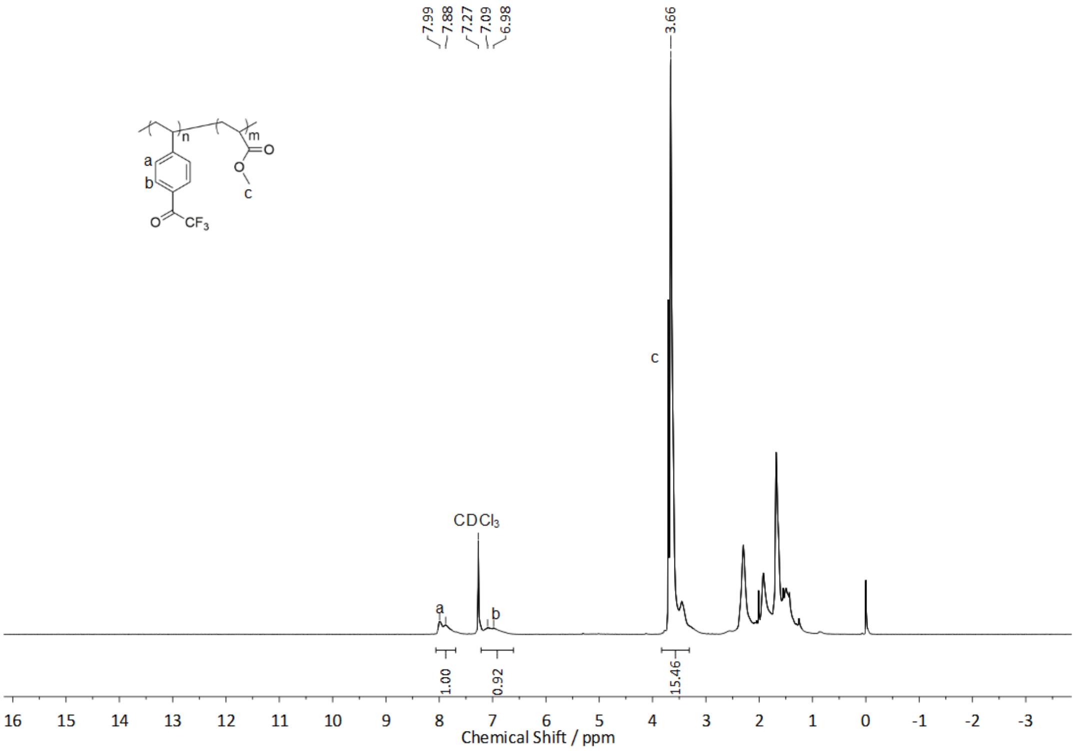

Supplementary Fig. 71 | ^{1}H NMR spectrum (in $CDCl_3$) of liner copolymer P(VTFP-MA)-10.

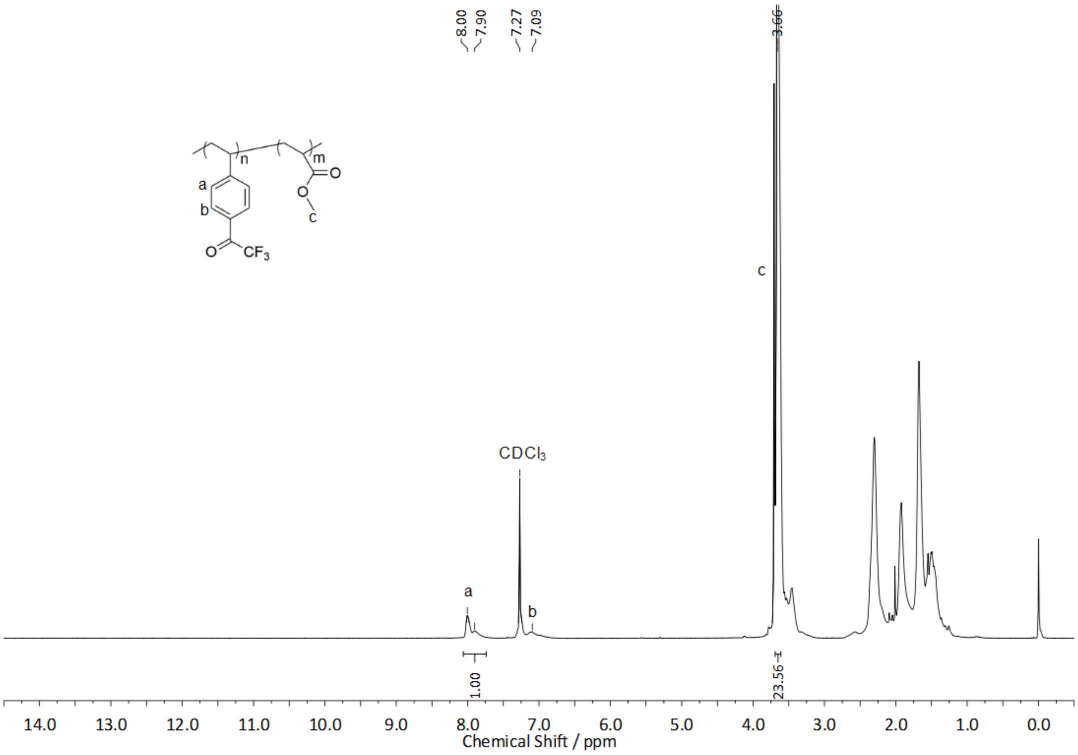

Supplementary Fig. 72 | ^{1}H NMR spectrum (in $CDCl_3$) of liner copolymer P(VTFP-MA)-15.

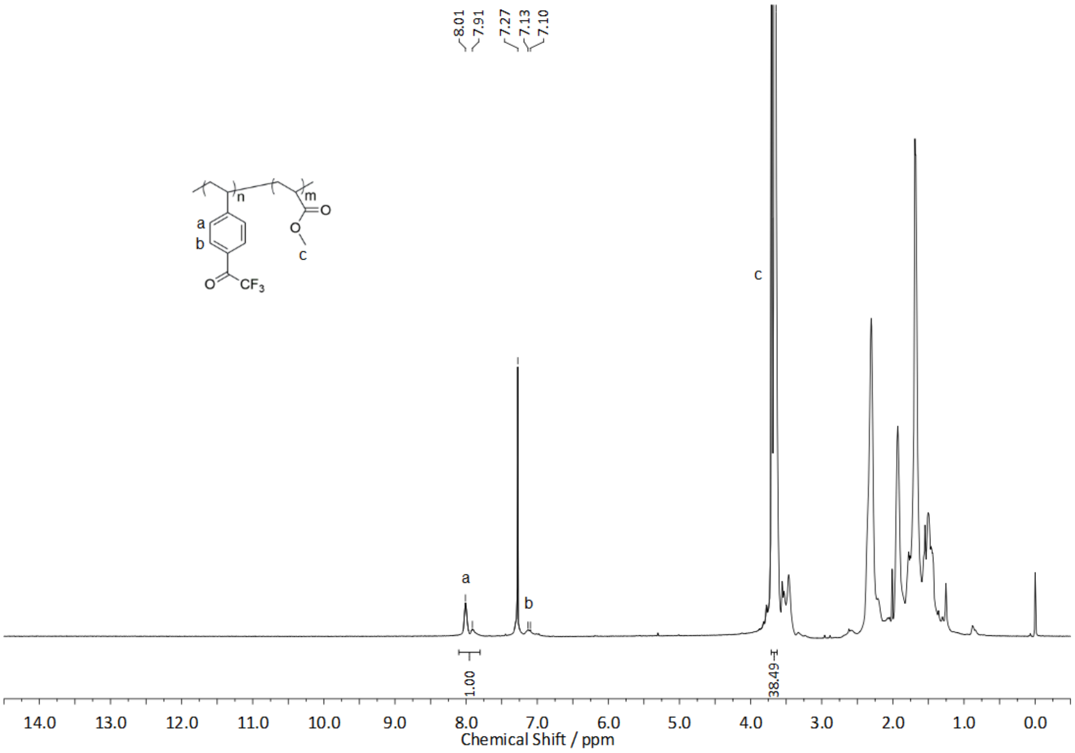

Supplementary Fig. 73 | ^{1}H NMR spectrum (in $CDCl_3$) of liner copolymer P(VTFP-MA)-25.

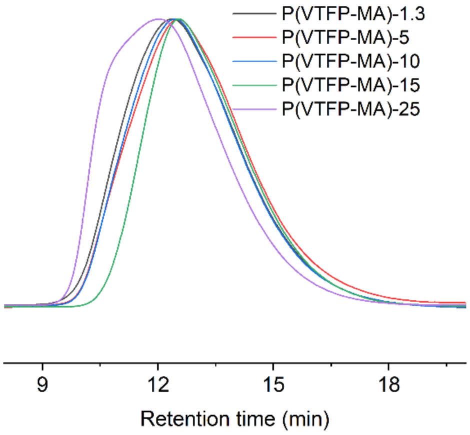

Supplementary Fig. 74 | GPC spectrum of liner copolymers P(VTFP-MA)-x with different monomer molar ratio of VTFP to MA, using THF as the eluent. P(VTFP-MA)-1.3: M_n = 48.8 kDa, $Đ$ = 1.84. P(VTFP-MA)- 5: M_n = 37.6 kDa, $Đ$ = 1.27. P(VTFP-MA)-10: M_n = 39.9 kDa, $Đ$ = 1.56. P(VTFP-MA)-15: M_n = 38.9 kDa, $Đ$ = 1.56. P(VTFP-MA)-25: M_n = 44.3 kDa, $Đ$ = 1.80.

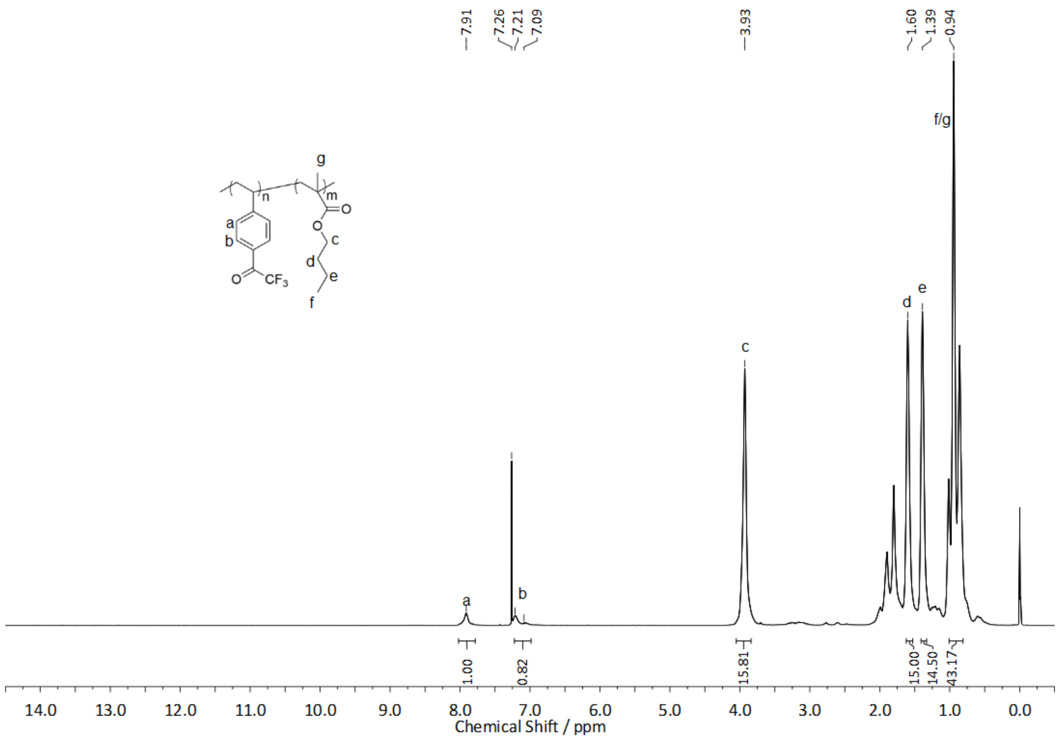

Supplementary Fig. 75 | ^{1}H NMR spectrum (in $CDCl_3$) of liner copolymer P(VTFP-BuMA)-15.

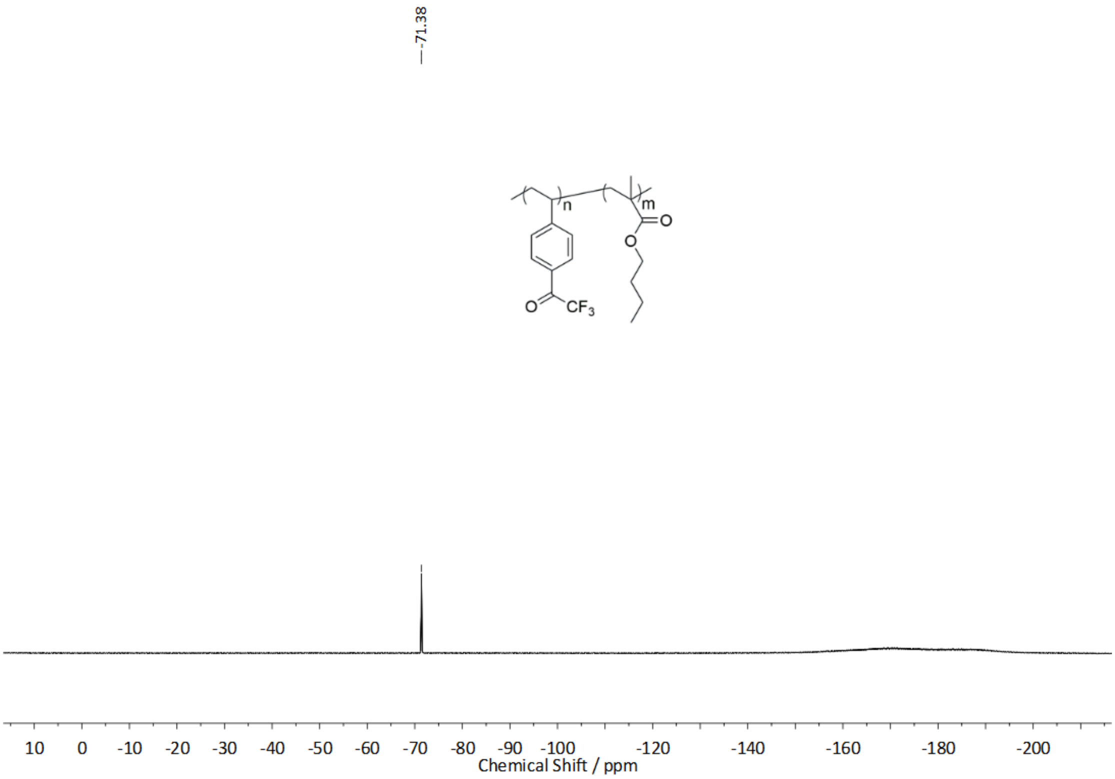

Supplementary Fig. 76 | ^{19}F NMR spectrum (in $CDCl_3$) of liner copolymer P(VTFP-BuMA)-15.

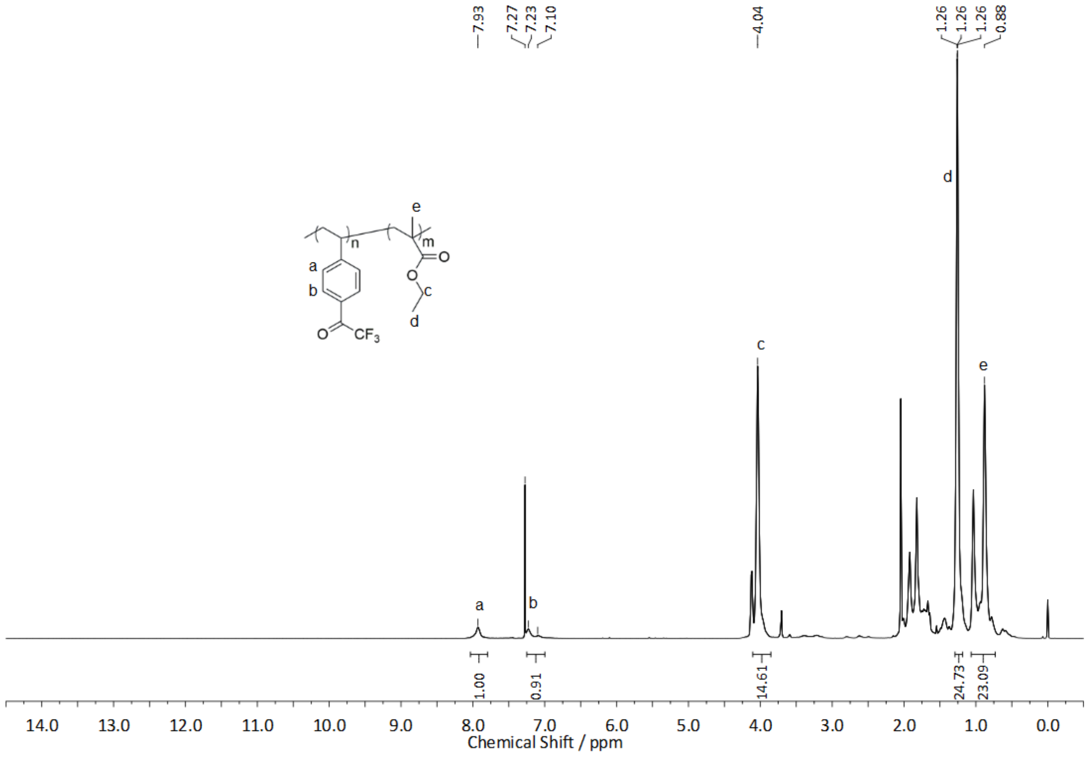

Supplementary Fig. 77 | ^{1}H NMR spectrum (in $CDCl_3$) of liner copolymer P(VTFP-EMA)-15.

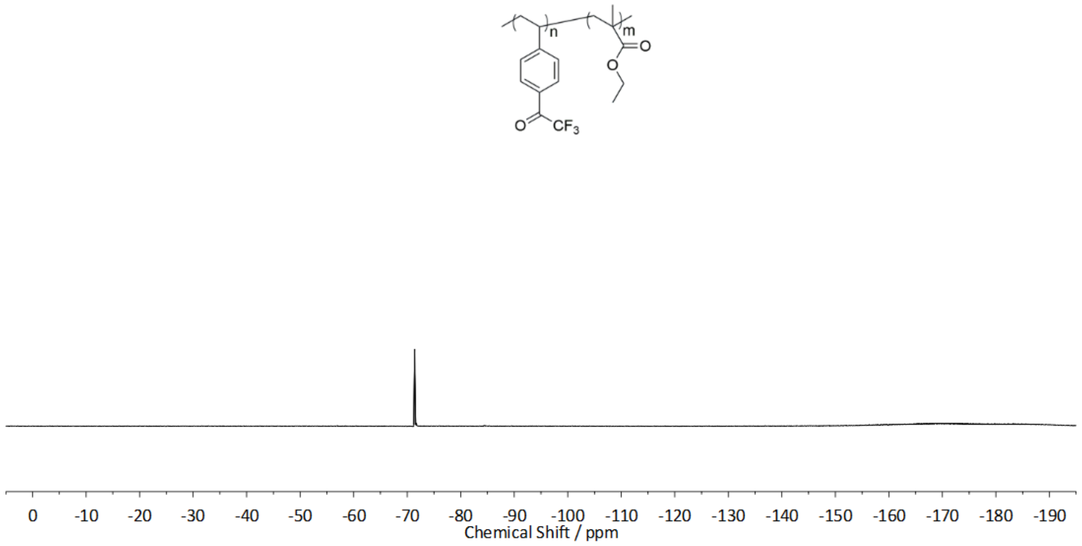

Supplementary Fig. 78 | ^{19}F NMR spectrum (in $CDCl_3$) of liner copolymer P(VTFP-EMA)-15.

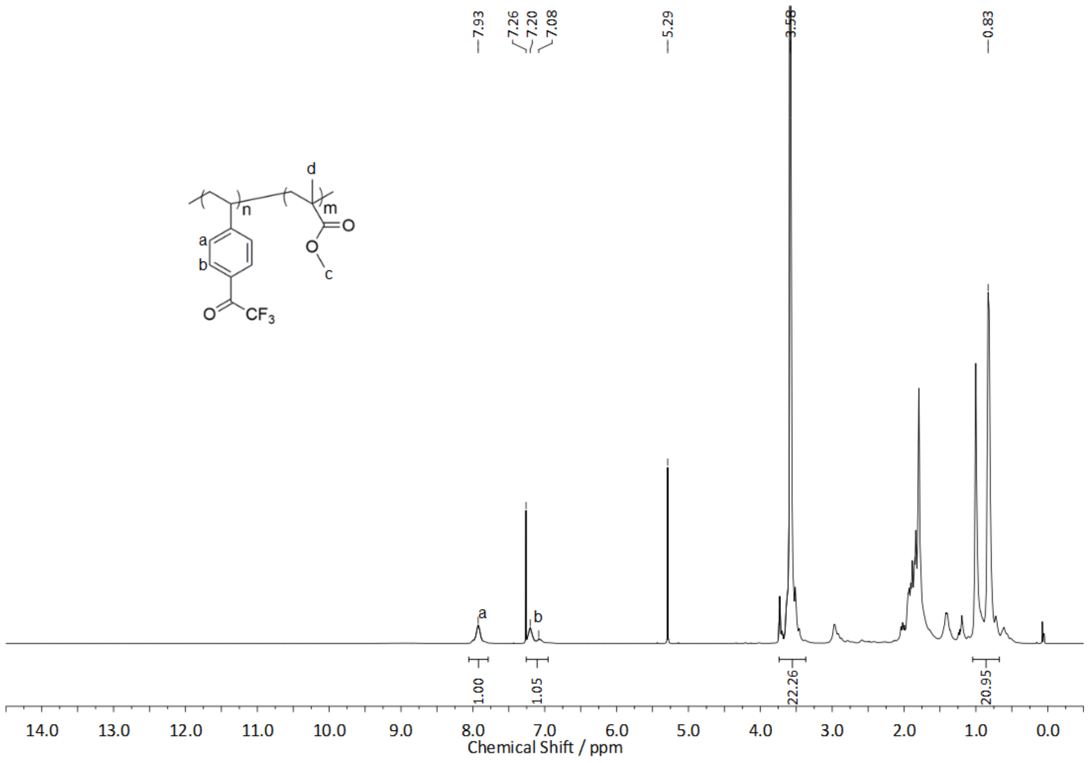

Supplementary Fig. 79 | ^{1}H NMR spectrum (in $CDCl_3$) of liner copolymer P(VTFP-MMA)-15.

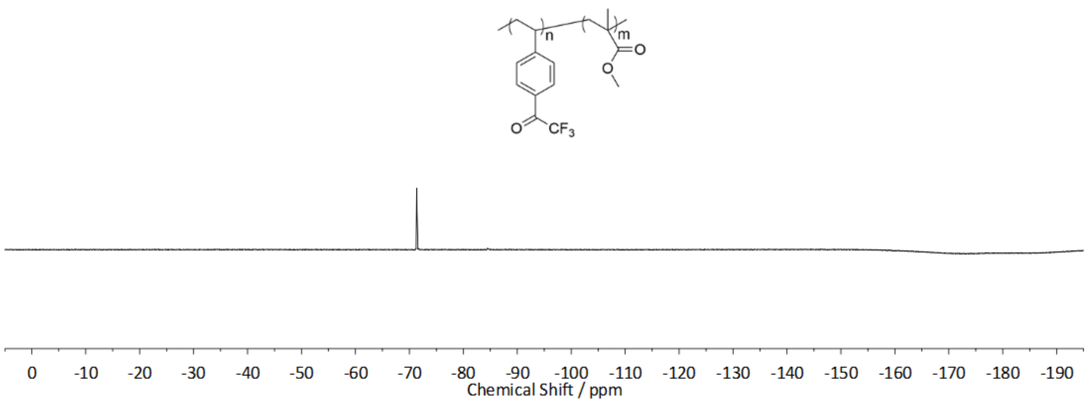

Supplementary Fig. 80 | ^{19}F NMR spectrum (in $CDCl_3$) of liner copolymer P(VTFP-MMA)-15.

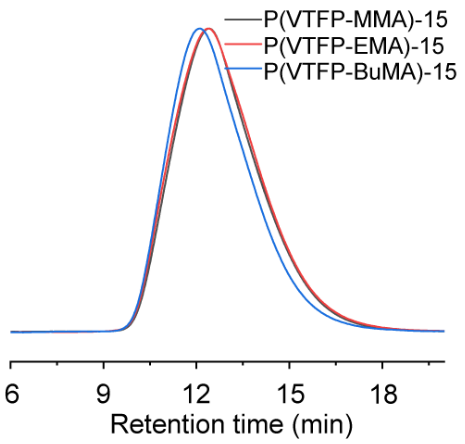

Supplementary Fig. 81 | GPC spectrum of TFP-containing copolymers with different comonomers, using THF as the eluent. P(VTFP-BuMA)-15: M_n = 80.7 kDa, $Đ$ = 1.29. P(VTFP-EMA)-15: M_n = 52.3 kDa, $Đ$ = 1.46. P(VTFP-MMA)-15: M_n = 59.4 kDa, $Đ$ = 1.45.

Supplementary Table 1 Comparative analysis of the CAN in this work with state-of-the-art CO_2-derived polymers

Type	CO_2 conversion strategy	CO_2 source	CO_2 content (%)	CO_2 conversion rate (%)	Synthesis conditions		Mechanical properties			Recyclability		Self-healing property	References
					Catalyst	Condition	Tensile strength (MPa)	Elongation at break (%)	Toughness (MJ/m^3)	Chemical Recyclability	Reprocessability		
This work	CO_3^{2-} /TFP dynamic coupling	ambient air	0.84-3.76	80.0	catalyst-free	RT, atmospheric pressure	4.3-38.9	0.7-937	0.019-68.17	0.5 M H_2SO_4, 30 min, ~95% efficiency	compression molding (80 °C, 8 MPa, 10 min); injection molding (100 °C)	80 °C, 0.5 MPa; 10 min; ~98.8% efficiency	-
epoxide-based polymers	CO_2/epoxide copolymerization	Pure CO_2	16.71-22.41	-	Cr(III)-PPN-F or zinc catalyst	1-2 MPa, 100 ℃, 24 h or RT	-	-	-	Cr(III)-PPN-F, 100 ℃, 10 min ~99% efficiency or TBD, 110 ℃, 16 h ~100% efficiency	-	-	1, 2
	CO_2/epoxide/diamine polymerization	Pure CO_2	10.77-11.21	-	Au/Fe_2O_3	1-2 MPa, 80-120 ℃, 8 h	18.45-37.81	275.22-691.25	41-125	-	-	-	3

Supplementary Table 1 (Continued.)

Type	CO_2 conversion strategy	CO_2 source	CO_2 content (%)	CO_2 conversion rate (%)	Synthesis conditions		Mechanical properties			Recyclability		Self-healing property	References
					Catalyst	Condition	Tensile strength (MPa)	Elongation at break (%)	Toughness (MJ/m^3)	Chemical Recyclability	Reprocessability		
olefins-based polymers	CO_2/olefin cyclization	Pure CO_2	11.62-28.52	-	$Pd(dba)_2$ and P(o-OMePh)$_3$	80 ℃, 20 h	-	-	-	3% ($Sn(Oct)_2$), 165 ℃, ~84% efficiency	-	-	4, 5
triple-bond monomer-based polymers	CO_2/alkyne/dihalides polymerization	Pure CO_2	15-24	-	Ag_2WO_4, Cs_2CO_3	80 ℃, 24 h	-	-	-	THF and NaOH aqueous solution, RT	-	-	6
	CO_2/alkyne cyclization	Pure CO_2	8.88-10.77	-	Ag(I)/Cu(I) catalysts, organic bases	90 ℃, 4 h	-	-	-	-	-	-	7
Frustrated Lewis Pair (FLP)-based polymers	CO_2/ FLP coupling	Pure CO_2	0.39-5.99	-	catalyst-free	RT, toluene	0.6-3.4	240-410	0.72-5.5	60 ℃ or 70 ℃, 2 h	-	swept by CO_2, 2 h; ~98% efficiency	8-10

Supplementary Table 2 Comparative analysis of the CAN in this work with CO_2-derived dynamic covalent networks

Dynamic covalent bonds	CO_2 source	CO_2 Content (%)	CO_2 conversion rate (%)	Synthesis conditions		Mechanical properties			Recyclability		Self-healing property	References
				Catalyst	Condition	Tensile strength (MPa)	Elongation at break (%)	Toughness (MJ/m^3)	Chemical Recyclability	Reprocessability		
This work (CO_3^{2-}-bridged bond)	ambient air	0.84-3.76	80	catalyst-free	RT, atmospheric pressure	4.3-38.9	0.7-937	0.019-68.17	0.5 M H_2SO_4, 30 min, ~95% efficiency	compression molding (80 °C, 8 MPa, 10 min); injection molding (100 °C)	80 °C, 0.5 MPa; 10 min; ~98.8% efficiency	-
N,S(O)-Acetal	Pure CO_2	8.96-13.82	-	Ag_2CO_3 and PPh_3 or CuI	25 °C, 4 MPa, 24 h or 60 °C, 80 atm, 24 h	1.3-70	0.7-159	0.25-9	200 °C, 2 min, triethylamine ~80% efficiency or 1 M MSA at 60 °C ~100% efficiency	compression molding (90-100 °C, 1-3 ton, 3-10 min); injection molding or extrusion (100 °C)	-	11-13

Supplementary Table 2 (Continued.)

Dynamic covalent bonds	CO_2 source	CO_2 Content (%)	CO_2 conversion rate (%)	Synthesis conditions		Mechanical properties			Recyclability		Self-healing property	References
				Catalyst	Condition	Tensile strength (MPa)	Elongation at break (%)	Toughness (MJ/m^3)	Chemical Recyclability	Reprocessability		
disulfide bond	Pure CO_2	6.2-10.86	-	TBAI and L-ascorbic acid or TBAB	100 °C, 3.5 MPa or 105 °C, atmospheric pressure, 24 h	1.5-7.7	120-358	1.3-8.95[3]	DTT, DMF, 60 °C, 30 min	compression molding (100 °C, 1 h or 150 °C, 1 MPa, 1 h)	1.50 °C or 80 °C; 2.UV healing （365 nm for 60 min） ~90% efficiency	14, 15
	dry ice	12.62-20.28	-	DBU, I_2	100 °C	0.2-19.1	1.2-203	0.17-0.9	-	compression molding (100 °C, 6 MPa, 20 min)	-	16
carbamate	Pure CO_2	6.45-11.4	-	TBAI and L-ascorbic acid or Cs_2CO_3	80-100 °C, 1-10 MPa, 24-48 h	0.71-103	4-280	0.37-16	glacial acetic acid and hydrogen peroxide, 60 °C, 4 h or 140 °C, ethanol, 5 h	compression molding (120-180 °C, 6-30 MPa, 20 min-8 h)	150 °C, 120 min or 120 °C, 3 h ~60% efficiency	17-20

Supplementary References

1. Liu, Y., Zhou, H., Guo, J. Z., Ren, W. M. & Lu, X. B. Completely Recyclable Monomers and Polycarbonate: Approach to Sustainable Polymers. *Angew. Chem. Int. Ed.* **56,** 4862–4866 (2017).
2. Li, C., Sablong, R. J., van Benthem, R. A. T. M. & Koning, C. E. Unique Base-Initiated Depolymerization of Limonene-Derived Polycarbonates. *ACS Macro Lett.* **6,** 684–688 (2017).
3. Li, S. et al. Crystallizable and Tough Aliphatic Thermoplastic Polyureas Synthesized through a Nonisocyanate Route. *Ind. Eng. Chem. Res.* **55,** 1902–1911 (2016).
4. Rapagnani, R. M., Dunscomb, R. J., Fresh, A. A. & Tonks, I. A. Tunable and recyclable polyesters from CO_2 and butadiene. *Nat. Chem.* **14,** 877–883 (2022).
5. Nakano, R., Ito, S. & Nozaki, K. Copolymerization of carbon dioxide and butadiene via a lactone intermediate. *Nat. Chem.* **6**, 325–331 (2014).
6. Song, B., He, B., Qin, A. & Tang, B. Z. Direct Polymerization of Carbon Dioxide, Diynes, and Alkyl Dihalides under Mild Reaction Conditions. *Macromolecules* **51,** 42–48 (2017).
7. Song, B., Li, X., Qin, A. & Tang, B. Z. Direct Conversion from Carbon Dioxide to Luminescent Poly(β-alkoxyacrylate)s via Multicomponent Tandem Polymerization-Induced Emission. *Macromolecules* **54,** 9019–9026 (2021).
8. Chen, L., Liu, R., Hao, X. & Yan, Q. CO_2-Cross-Linked Frustrated Lewis Networks as Gas-Regulated Dynamic Covalent Materials. *Angew. Chem. Int. Ed.* **58,** 264–268 (2018).
9. Liu, R., Liu, X., Ouyang, K. & Yan, Q. Catalyst-Free Click Polymerization of CO_2 and Lewis Monomers for Recyclable C1 Fixation and Release. *ACS Macro Lett.* **8,** 200–204 (2019).
10. Liu, R., Wang, Y. & Yan, Q. CO_2-Strengthened Double-Cross-Linked Polymer Gels from Frustrated Lewis Pair Networks. *Macromol. Rapid Commun.* **42,** 2000699–2000705 (2021).
11. Habets, T. et al. Covalent Adaptable Networks through Dynamic N,S-Acetal

Chemistry: Toward Recyclable CO_2-Based Thermosets. *J. Am. Chem. Soc.* **145,** 25450–25462 (2023).

12. Caliari, M. et al. Fully Recyclable Pluripotent Networks for 3D Printing Enabled by Dissociative Dynamic Bonds. *Adv. Mater.* **37,** 2417355–2417367 (2025).
13. Caliari, M. et al. A Material Platform Based on Dissociative CO_2-Derived N,O-Acetals for Tunable Degradation of 3D Printable Materials. *J. Am. Chem. Soc.* **147,** 30095–30106 (2025).
14. Yang, X. et al. Preparation of non-isocyanate polyurethanes from epoxy soybean oil: dual dynamic networks to realize self-healing and reprocessing under mild conditions. *Green. Chem.* **23,** 6349–6355 (2021).
15. Thakur, S., Verdejo, R. & Angel Lopez Manchado, M. Unveiling the potential of self-healing and closed-loop recyclability in bio-based non-isocyanate polyurethane. *Eur Polym J.* **217,** 113296–113305 (2024).
16. Pronoitis, C., Hakkarainen, M. & Odelius, K. Structurally Diverse and Recyclable Isocyanate-Free Polyurethane Networks from CO_2-Derived Cyclic Carbonates. *ACS Sustainable Chem. Eng.* **10,** 2522–2531 (2022).
17. Seychal, G. et al. Synergetic Hybridization Strategy to Enhance the Dynamicity of Poorly Dynamic CO_2-derived Vitrimers achieved by a Simple Copolymerization Approach. *Adv. Funct. Mater.* **35,** 2412268–2412280 (2024).
18. Ge, W., Zhao, B., Li, L., Nie, K. & Zheng, S. Nanocomposites of polyhydroxyurethane with nanocrystalline cellulose: Synthesis, thermomechanical and reprocessing properties. *Eur. Polym. J.* **149,** 110287–110288 (2021).
19. Yang, X. et al. Recyclable non-isocyanate polyurethanes containing a dynamic covalent network derived from epoxy soybean oil and CO_2. *Mater. Chem. Front.* **5,** 6160–6170 (2021).
20. Baek, S., Lee, J., Kim, H., Cha, I. & Song, C. Self-Healable and Recyclable Biomass-Derived Polyurethane Networks through Carbon Dioxide Immobilization. *Polymers* **13,** 4381–4395 (2021).
