# Supplementary material for: Upcycling of atmospheric CO2 to self-healing recyclable polymers under ambient conditions
Source: Nat Commun. 2026 Mar 2;17:3349. doi: 10.1038/s41467-026-70046-6 (PMC13066383; doi:10.1038/s41467-026-70046-6)
Supplement: Supplementary file 2 — Description of Additional Supplementary File [file 41467_2026_70046_MOESM2_ESM.pdf]

The Description of Additional Supplementary Files

Supplementary Movie 1. The detection of CO_2 as a product in the model compound. As the reaction proceeded under ambient conditions, vigorous gas evolution was observed, accompanied by a distinct opacification process of the initially transparent $Ca(OH)_2$ aqueous solution. This indicates that the gas formed is CO_2.

Supplementary Movie 2. Load-bearing capacity of repaired CO_2-derived CAN. The repaired sample supported a 1 kg object weighing over 5,000 times its own weight for at least 5 min at ambient temperature, with no significant extension.

Supplementary Movie 3. Tensile test of repaired CO_2derived CAN.
